# Supplementary figures and images for: Disseminated Intravascular Coagulation (DIC): Old player creates new perspectives on the polymicrobial sepsis model of CASP
Source: PLoS One. 2022 Dec 8;17(12):e0277492. doi: 10.1371/journal.pone.0277492 (PMC9731468; doi:10.1371/journal.pone.0277492)

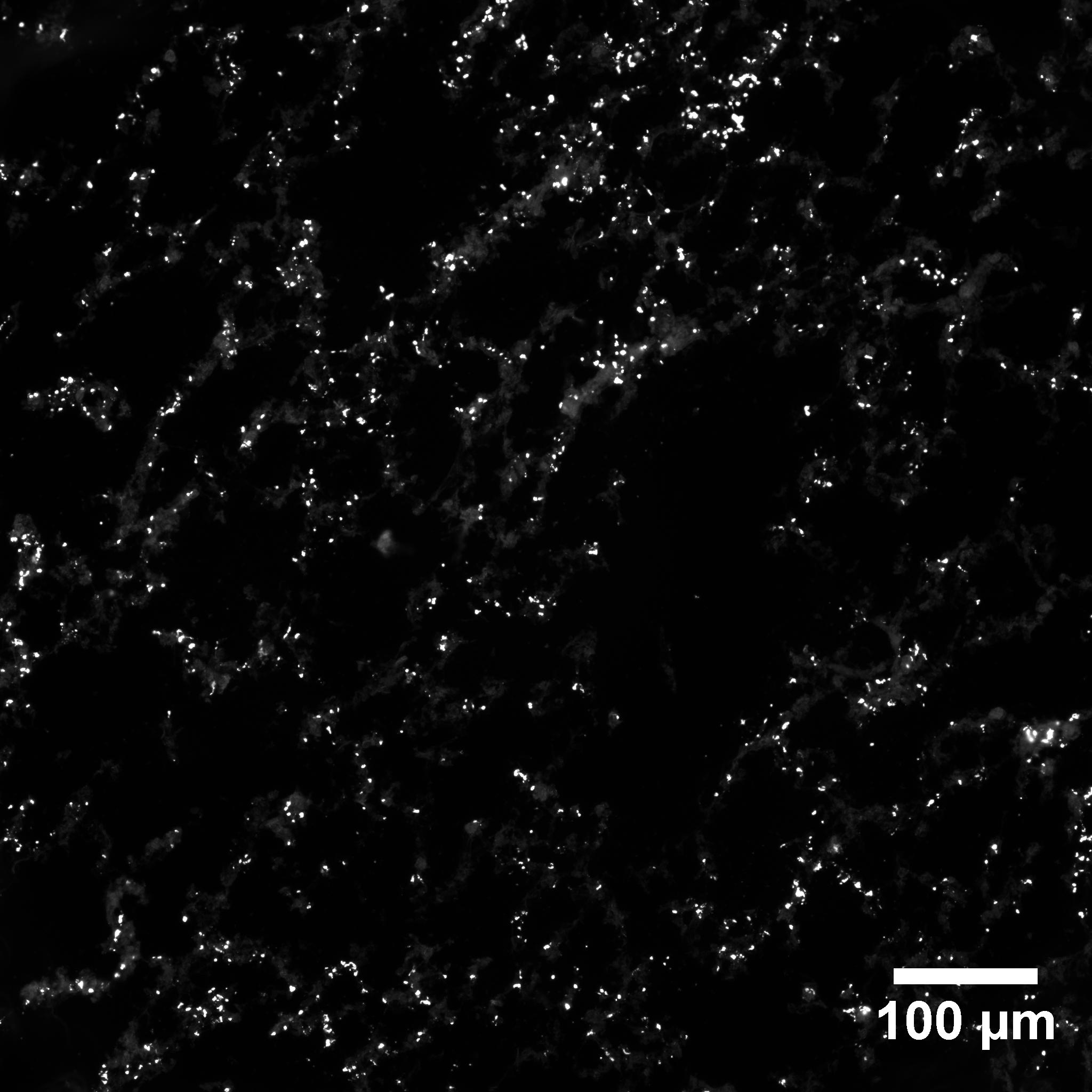

Supplement: S1 File — (ZIP) [file pone.0277492.s001.zip › PACE Corrected/LungeWTKO1_20x_2sec_S1c.tif]

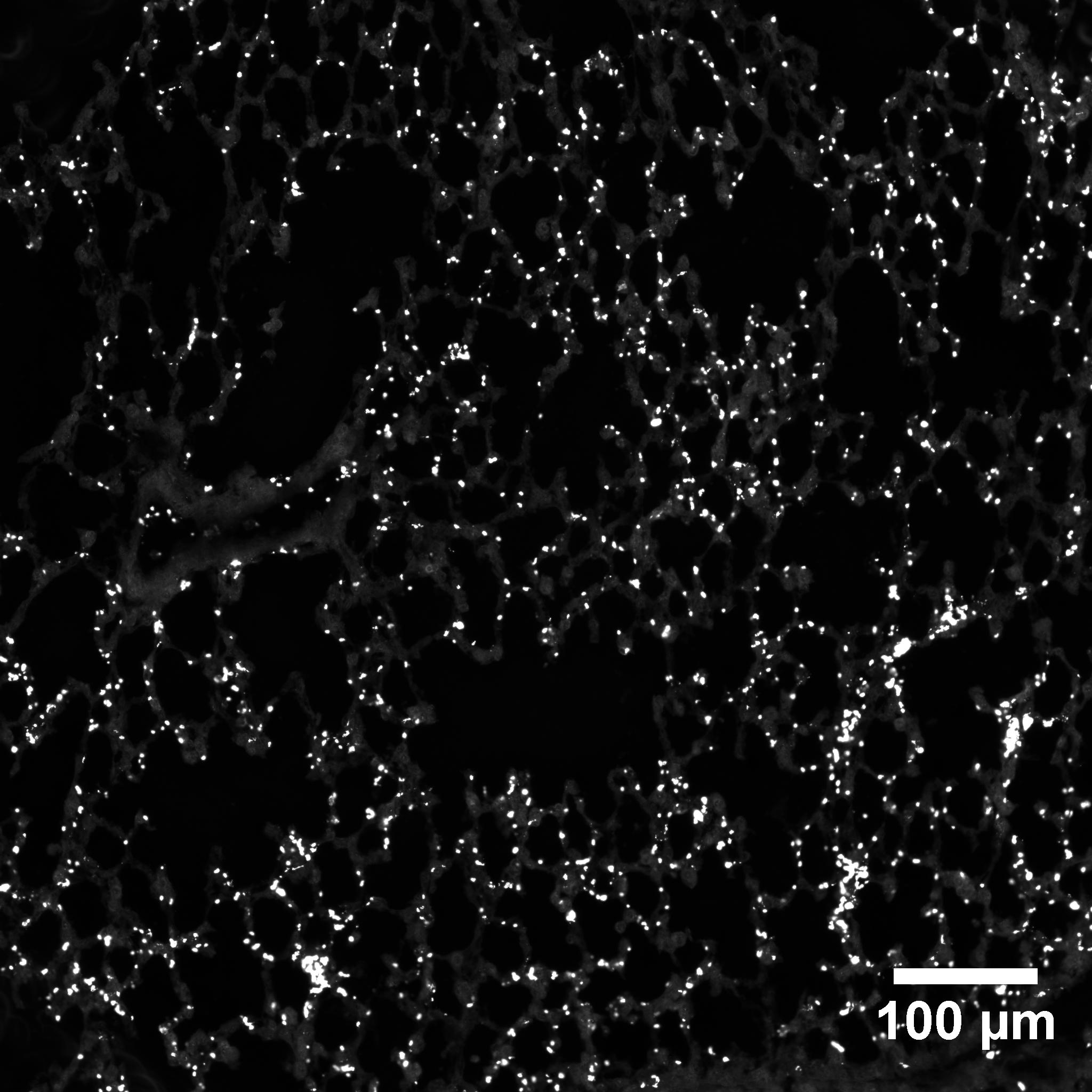

Supplement: S1 File — (ZIP) [file pone.0277492.s001.zip › PACE Corrected/LungeWTCASP1_20x_2sec_S1d.tif]

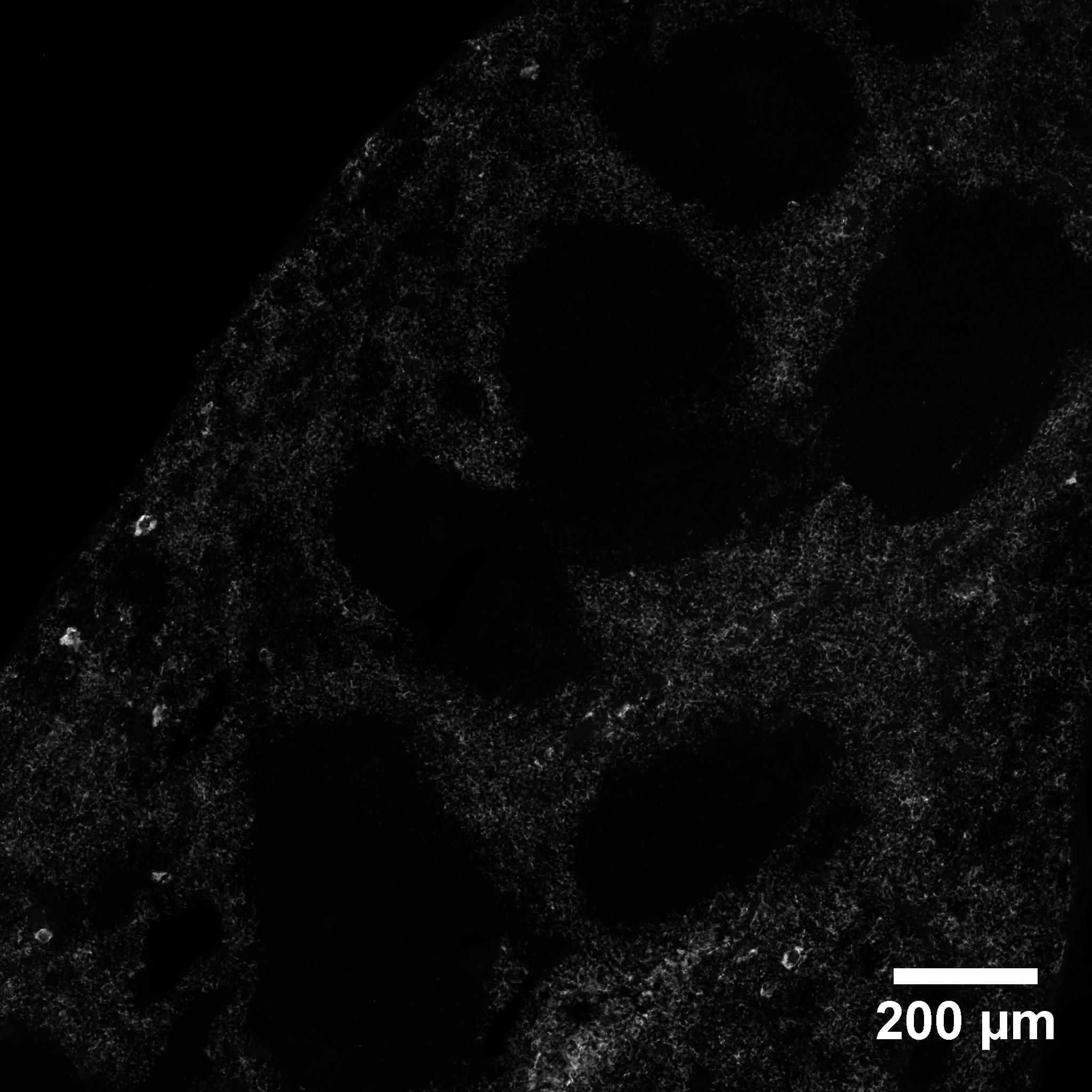

Supplement: S1 File — (ZIP) [file pone.0277492.s001.zip › PACE Corrected/WTKontrolleMilz1_10x_1sec_S1a.tif]

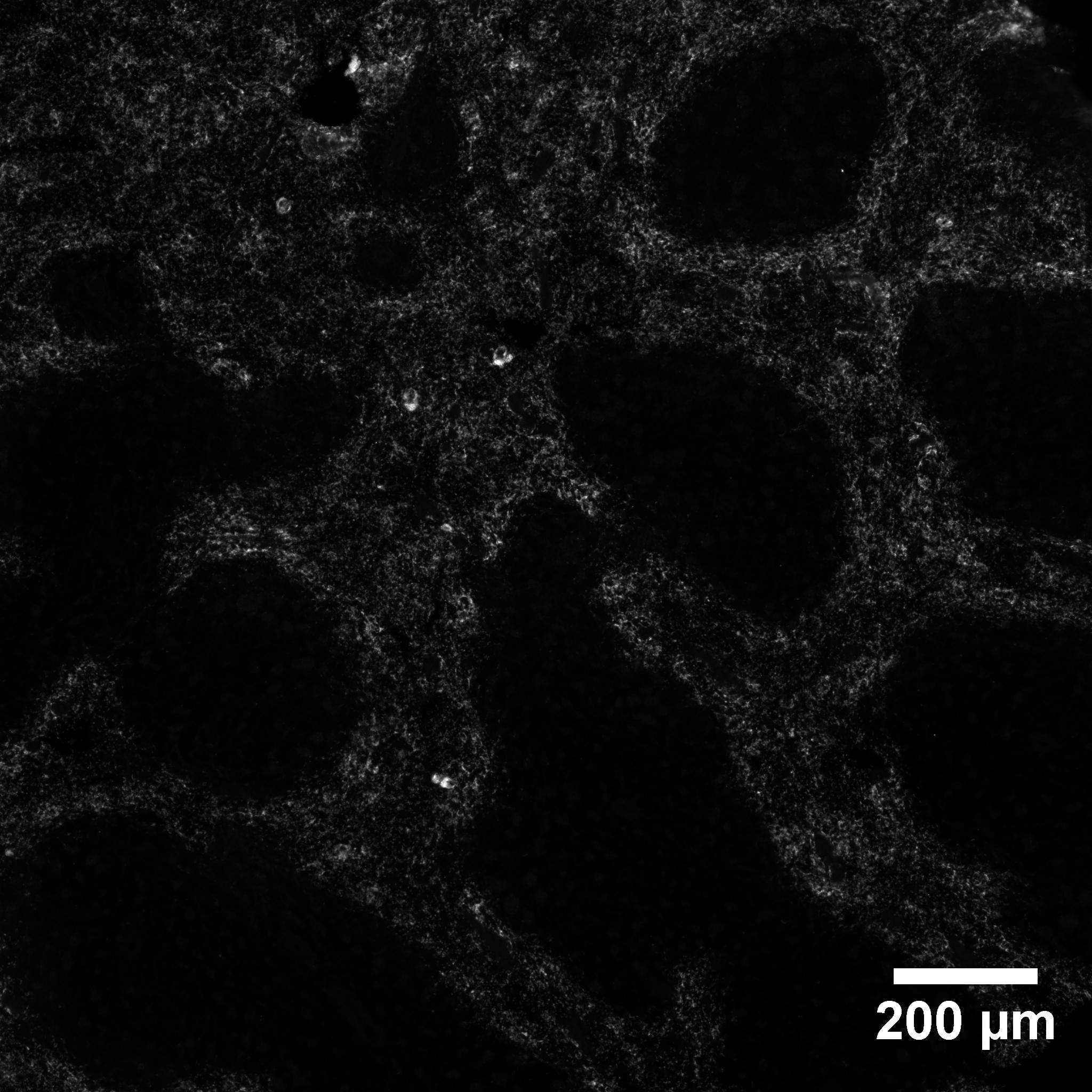

Supplement: S1 File — (ZIP) [file pone.0277492.s001.zip › PACE Corrected/WTCASPMilz1_10x_1sec_S1b.tif]

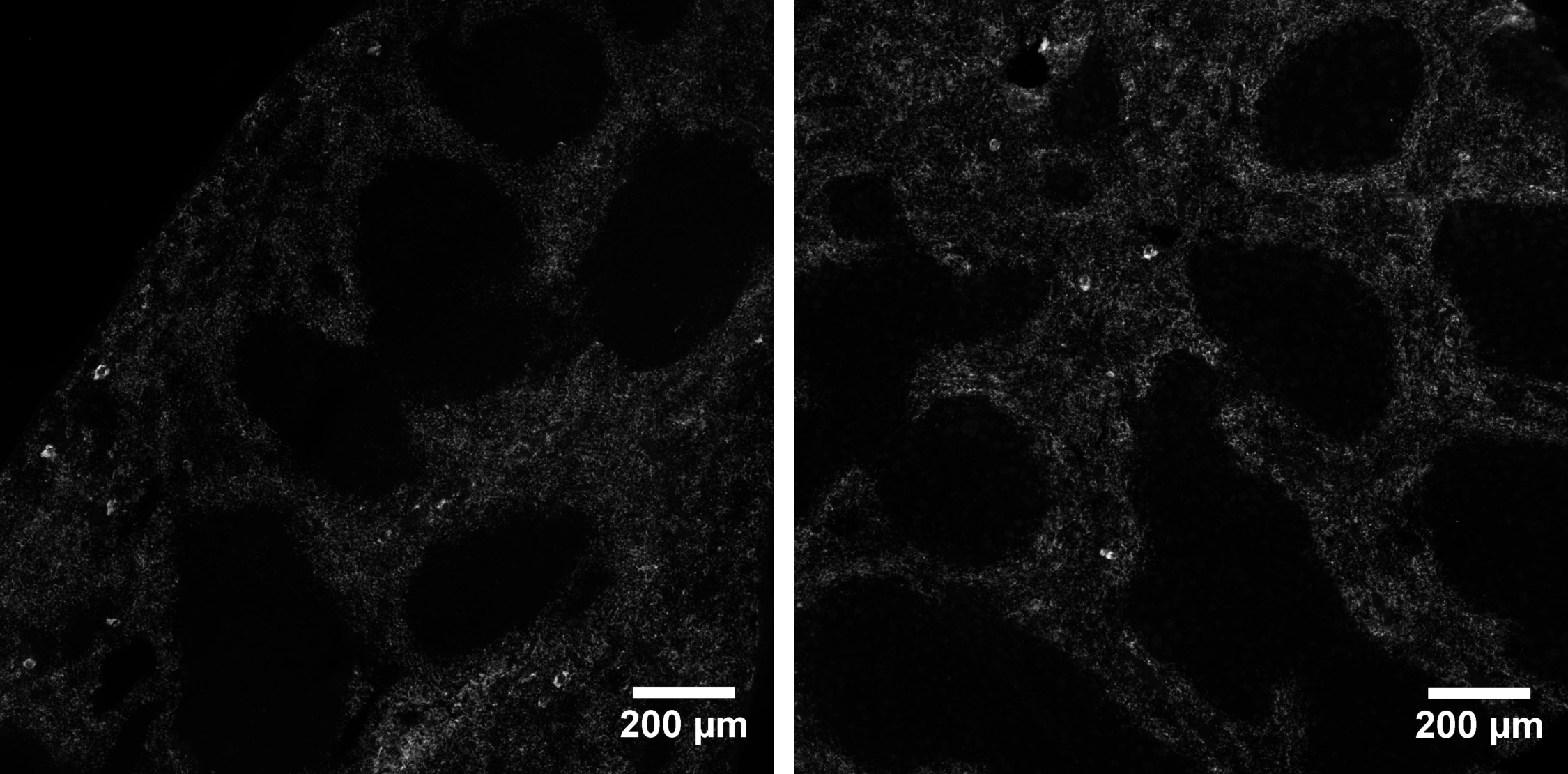

Supplement: S1 Fig — Immunohistochemical staining of frozen spleen sections 20 h after CASP with anti-CD42c*FITC/Alexa 488. Depicted are representative results of frozen sections from 8- to 10-week-old C57Bl/6 organ tissue with immunohistochemistry staining of platelets with anti-CD 42c antibody*FITC/Alexa 488. The sections show distribution of platelets in spleen tissue: untreated mice (left figure) and CASP operated (right figure) (scale bar in μm). (TIF) [file pone.0277492.s002.tif]

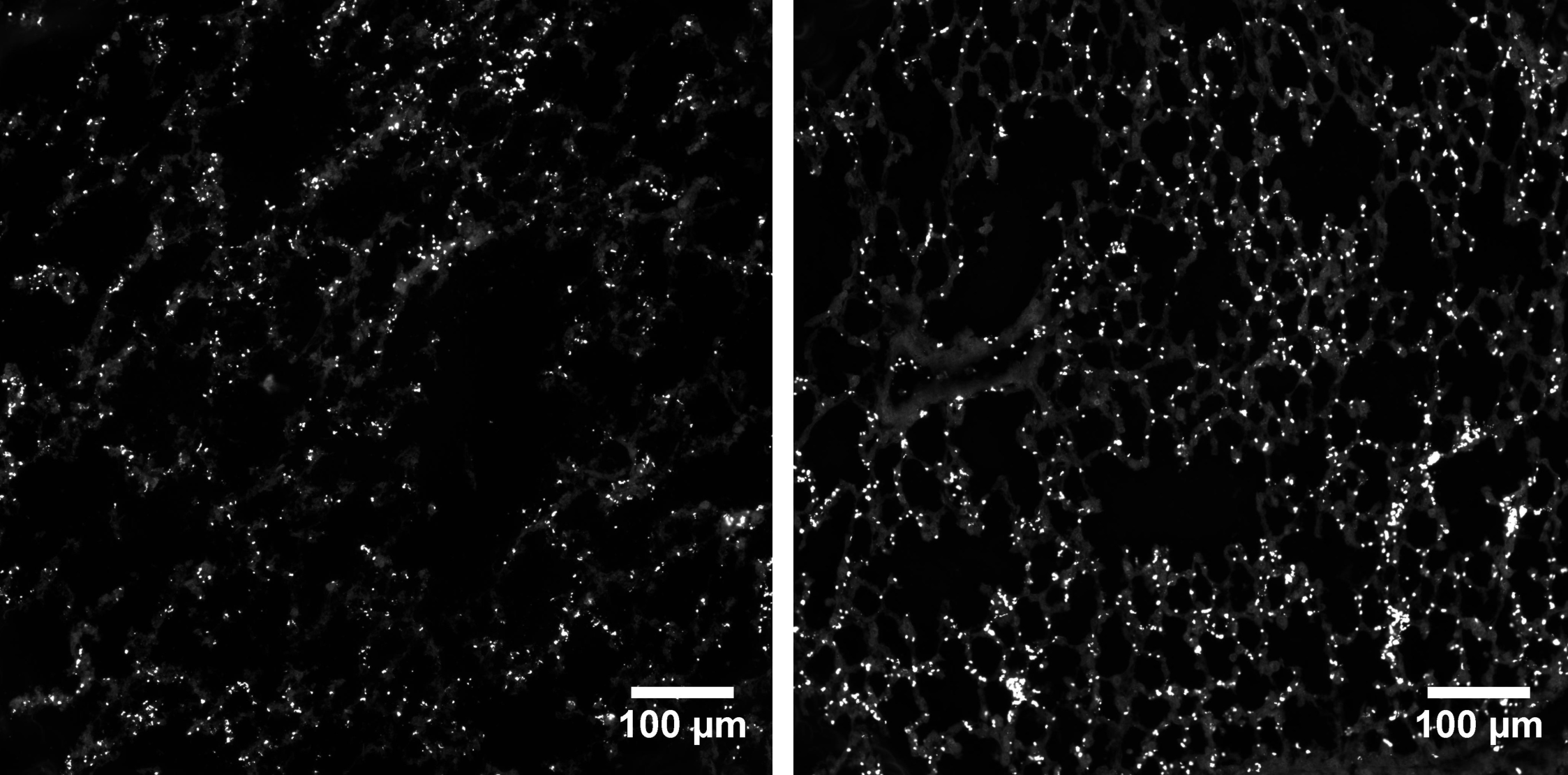

Supplement: S2 Fig — Immunohistochemical staining of frozen lung sections 20 h after CASP with anti-CD42c*FITC/Alexa 488. Depicted are representative results of frozen sections from 8- to 10-week-old C57Bl/6 organ tissue with immunohistochemistry staining of platelets with anti-CD 42c antibody*FITC/Alexa 488. The sections show distribution of platelets in lung tissue: untreated mice (left figure) and CASP operated (right figure) (scale bar in μm). (TIF) [file pone.0277492.s003.tif]
