# Supplementary material for: Disseminated Intravascular Coagulation (DIC): Old player creates new perspectives on the polymicrobial sepsis model of CASP
Source: PLoS One. 2022 Dec 8;17(12):e0277492. doi: 10.1371/journal.pone.0277492 (PMC9731468; doi:10.1371/journal.pone.0277492)
Supplement: Attachment — Submitted filename: PONE-D-22-09810 response to reviewers rebutal letter 260922.docx [file pone.0277492.s004.docx]

PONE-D-22-09810

Disseminated Intravascular Coagulation (DIC): Old player creates new perspectives on the polymicrobial sepsis model of CASP.

Dear Dr. Elizabeth S. Mayne,
Thank you for sending us a revision of our manuscript. We would also like to thank the reviewers for suggesting improvements to this work. We tried to answer the individual points conscientiously and changed the illustrations accordingly. Unfortunately, the endnote program doesn't play along with the square brackets today. I will try to fix it.

We look forward to your answer. Stay healthy and safe.

Kind regards,

Julia van der Linde

Department General Surgery

University medicine Greifswald

Reviewer #1: This manuscript describes the coagulation profile of a mouse model of polymicrobial intra-abdominal sepsis (CASP), and demonstrates potential as a reliable animal model for further research on the pathogenesis and management of disseminated intravascular coagulation. It is overall well written, but requires some modification:

1) The data analysis has largely been performed on very small numbers of mice, and does not appear to be likely to be normally distributed. As such, the mean results reported may be skewed due to the impact of one or two outliers (particularly in the case of the ROTEM data and the bleeding times). Median and interquartile ranges should be reported instead of means and standard deviations. With these results, it will be easier to determine if all of the coagulation parameters are indeed significantly different in the CASP mice.

*Thank you for your comments, we have endeavored to adapt the charts and present them with the median values and interquartile measurements accordingly.*

2) All of the experiments were not performed on all of the mice. This was explained in the discussion, but should also be mentioned in the methods, along with the overall number of mice tested (including the controls).

*We added comments on this in the part of Materials and Methods Line 118-127.*

3) In the introduction, the inflammasome is referred to in line 72. A brief explanation of what the inflammasome is would be of value.

*Thank you for this note. An explanation was added in Line 84-96, in which we noticed that the described pathophysiology of the inflammasome is even more applicable to the CASP model, since there is a massive release of interleukin 1ß, as could be shown in earlier data. (Maier et al 2004.)*

4) In line 82, it is stated that “Bacteremia is vital for developing DIC,”. This is not correct, as many non-infectious causes of DIC are known (as mentioned previously in the introduction).

*In line 94 we added: …” In these models, dealing with bacterial pathogens,…”*

5) In line 86, the meaning of the phrase “with a high yielding production” is unclear.

In line 99 we changed to: *…”repetitious accuracy”…*

6) The manufacturer along with the manufacturers city and country should be specified for all kits and instruments used (including the Vetscan, the ROTEM, and the D-Dimer and Fibrinogen kits). *Changed in Line:*

*128 BD Venflon, Franklin Lakes, USA*

*142 Vetscan Abaxis Union City, USA*

*158 ROTEM (TEM international Gmbh, Munich, Germany)*

*165 Stago Asserachrom (Asnieres sur Seine, France)*

*170 Abnova Taipei, Taiwan*

*195 Keyence, Osaka, Japan*

7) In line 128-130, the meaning of the following sentence: “Data from two experiments were pooled to minimize the time between blood sampling and analysis of the probes in ROTEM® delta.” is unclear.

*For the coagulation analysis, the time from blood collection to analysis of the samples should be as short as possible, since the ROTEM can only analyze 4 samples at the same time. the results from 2 experimental days were collected and summarized.*

*Linie 159-161: We changed the sentence to the following:…” Because the analyzer can only evaluate 4 samples at the same time, data from two experimental days were collected to minimize the time between blood sampling and analysis of the probes in ROTEM® delta.”…*

8) For all the results, the number of included mice is given as a range (eg n=5-8 or n=23-25). Why is this? Please explain in the methods.

*Thank you for your advice: The different group sizes are due to the complex methods used. On the one hand, the animals in the CASP-operated group might had a Stress Severity Score of over 10 and where released from the experiments prematurely, on the other hand, in our experience blood sampling is more difficult in septic animals, this fact was taken into account when choosing a larger group size of CASP.*

*The normal coagulation parameters of C57Bl/6 are well known, so that a large control group could be dispensed with, provided that the experimental method itself does not pose large variables.*

*We changed the ranges and explained it separately.*

9) In line 245, “HE staining of the liver” is mentioned. What is this? Please include in the methods.

*Included in Line 203-212* *Results are not shown*
10) In line 254, the FITC positive area is reported. How is this calculated? Please include in the methods.

*Line 202: …“The TRITC positive area was analyzed with ImageJ software.”…*

Minor grammatical corrections:

1) In line 16 it is stated that “In surgical ICUs, DIC is mainly caused by abdominal sepsis”. Please rephrase this as follows: “In surgical ICUs, DIC is frequently caused by abdominal sepsis” **Done**
2) In lines 45-47, the following: “The complexity and necessity of understanding coagulation disorders in the critically ill became highlighted during COVID-19 (3). This observation illustrates the significance of coagulation’s influence on the prognosis of critically ill patients.” Should be rephrased as follows: “The complexity and necessity of understanding coagulation disorders in the critically ill was highlighted during the COVID-19 pandemic (3), where the influence of coagulation abnormalities on the prognosis of critically ill patients was illustrated.” **Done; Line 46-48**
3) In line 50, I suggest removing “micro-organisms” from the list of causes of DIC. **Done; in Line 57**
4) In line 58, please change “increase the intake of platelets” to “increase the consumption of platelets”. **Done; Line 66**
5) The sentence in lines 258-261 should be rephrased as follows: “The double staining of fibrinogen and platelets in CASP sepsis clearly verified increased fibrinogen in the area of the sinusoids in untreated mice, while fibrinogen was less readily seen in those with sepsis.” **Done; in Line 426-428**

Reviewer #2: With this manuscript, the authors showed that the CASP model is suitable for analysing the complexity of DIC in polymicrobial sepsis. The experiments were performed on 8-10 weeks old female C57BL/6 mice, but the number of animals used was not mentioned in the methodology section. Treated and non-treated animals were compared in terms of platelet count, bleeding time, TEG, D-Dimer, Fibrinogen and immunohistological staining for platelets and fibrinogen. Again, the number of blood samples used for these tests were not mentioned.

*We added the exact numbers.*
The authors found that the CASP model caused thrombocytopenia, elongation of bleeding time, reduction in fibrinogen levels and the presence of D-Dimers in plasma. They mentioned that the CASP model seems superior to other models for inducing DIC, however they did not compare the different models directly. They should rather stated that the CASP model is suitable to analyse the complexity of DIC in polymicrobial sepsis.

*Thanks for your comments, we completed in line 590*

*“All in all CASP model is suitable to analyze the complexity of DIC in polymicrobial sepsis.”
This paper is furthermore well-written and recommended for publication.*

In Line 573 we added: The funders had no role in study design, data collection and analysis, decision to publish, or preparation of the manuscript.
