# Supplementary material for: Disseminated Intravascular Coagulation (DIC): Old player creates new perspectives on the polymicrobial sepsis model of CASP
Source: PLoS One. 2022 Dec 8;17(12):e0277492. doi: 10.1371/journal.pone.0277492 (PMC9731468; doi:10.1371/journal.pone.0277492)
Supplement: Attachment — Submitted filename: PONE-D-22-09810_R1 comments.docx [file pone.0277492.s005.docx]

PLOS ONE

#### Disseminated Intravascular Coagulation (DIC): Old player creates new perspectives on the polymicrobial sepsis model of CASP.

--Manuscript Draft--

| Manuscript Number: | PONE-D-22-09810R1 |
| --- | --- |
| Article Type: | Research Article |
| Full Title: | Disseminated Intravascular Coagulation (DIC): Old player creates new perspectives on the polymicrobial sepsis model of CASP. |
| Short Title: | Disseminated Intravascular Coagulation in sepsis model. |
| Corresponding Author: | Julia van der Linde, M.D.  University of Greifswald Faculty of Medicine: Universitatsmedizin Greifswald Greifswald, GERMANY |
| Keywords: | sepsis; DIC; Colon Ascendens Stent Peritonitis; animal model |
| Abstract: | Background  Disseminated intravascular coagulation (DIC) is a life-threatening complication of sepsis. In surgical ICUs, DIC is mainly caused by abdominal sepsis, and the disarranged coagulation and complications often lead to death. The severity of sepsis is associated with a higher DIC score according to the parameters proposed by the International Society of Hemostasis and Thrombosis (ISTH) in 2001: platelet count, bleeding time (Quick), D-dimer, and fibrinogen. One problem in studying DIC is finding an adequate animal model that reflects the clinical situation of polymicrobial overwhelming infection.  Aims and Methods  We investigated whether a well-established polymicrobial sepsis model of colon ascendens stent peritonitis (CASP) is suited to investigate the complexity of DIC. For this purpose, CASP-operated mice were examined 20 h after the operation with regard to coagulation parameters using cell counts, bleeding times, rotational thromboelastometry (ROTEM), ELISAs for D-dimer and fibrinogen, and platelet accumulation in affected organs via immunohistochemistry to see if the mice develop a coagulation disorder that meets the definition of DIC proposed by the ISTH 2001 consensus conference.  Results  Herein, we showed that the CASP model is an all-encompassing animal model to analyze the complexity of systemic DIC in murine abdominal sepsis. There is highly reproducible thrombocytopenia, a significant prolongation of the bleeding time, and a loss of fibrinogen in plasma. We also observed microvascular thrombosis due to platelet accumulation in the microcirculation of the liver.  Conclusion  The CASP model seems superior to other artificial models, e.g., injecting substances, for inducing DIC. CASP is one of the best true-to-life models for analyzing the complexity of disseminated intravascular coagulation in polymicrobial sepsis. |
| Order of Authors: | Julia van der Linde, M.D. |
|  | Stephan Diedrich, M.D. |
|  | Thorben Klee, M.D. |
|  | Claus-Dieter Heidecke, Prof. |
|  | Stephan Kersting, Prof. |
|  | Wolfram Keßler, P.D. |

*Powered by Editorial Manager® and ProduXion Manager® from Aries Systems Corporation*

| Response to Reviewers: | Dear reviewer no1  Thank you for your comments, we appreciate your effort to us and have endeavored to adapt the charts and present them with the median values and interquartile measurements accordingly. We added every single note conscientiously.  Thank you for your advice: The different group sizes are due to the complex methods used. On the one hand, the animals in the CASP-operated group might had a Stress Severity Score of over 10 and where released from the experiments prematurely, on the other hand, in our experience blood sampling is more difficult in septic animals, this fact was taken into account when choosing a larger group size of CASP.  The normal coagulation parameters of C57Bl/6 are well known, so that a large control group could be dispensed with, provided that the experimental method itself does not pose large variables.  We changed the ranges and explained it separately.  Dear reviewer no2  Thank you for your statement and comments, we completed in line 590  “All in all CASP model is suitable to analyze the complexity of DIC in polymicrobial sepsis.”  Best wishes to all kind regards  Julia van der Linde |
| --- | --- |
| Additional Information: |  |
| Question | Response |
| Financial Disclosure  Enter a financial disclosure statement that describes the sources of funding for the work included in this submission. Review the [submission guidelines](http://journals.plos.org/plosone/s/submission-guidelines#loc-financial-disclosure-statement) for detailed requirements. View published research articles from [*PLOS ONE*](http://journals.plos.org/plosone/) for specific examples.  This statement is required for submission and will appear in the published article if the submission is accepted. Please make sure it is accurate. | YES - This work was financially supported by the German Research Foundation (RTG 840; grants to JvdL 2009). |

*Powered by Editorial Manager® and ProduXion Manager® from Aries Systems Corporation*

| Unfunded studies  Enter: *The author(s) received no specific funding for this work.*  Funded studies  Enter a statement with the following details:   - Initials of the authors who received each award - Grant numbers awarded to each author - The full name of each funder - URL of each funder website - Did the sponsors or funders play any role in the study design, data collection and analysis, decision to publish, or preparation of the manuscript? - NO - Include this sentence at the end of   your statement: *The funders had no role in study design, data collection and analysis, decision to publish, or preparation of the manuscript.*   - YES - Specify the role(s) played. |  |
| --- | --- |
| * typeset |  |
| Competing Interests  Use the instructions below to enter a competing interest statement for this submission. On behalf of all authors, disclose any [competing interests](http://journals.plos.org/plosone/s/competing-interests) that could be perceived to bias this work—acknowledging all financial support and any other relevant financial or non­financial competing interests.  This statement is required for submission and will appear in the published article if the submission is accepted. Please make sure it is accurate and that any funding sources listed in your Funding Information later in the submission form are also declared in your Financial Disclosure statement.  View published research articles from  [*PLOS ONE*](http://journals.plos.org/plosone/) for specific examples. | NO - The authors have declared that no competing interests exist. |

*Powered by Editorial Manager® and ProduXion Manager® from Aries Systems Corporation*

| NO authors have competing interests  Enter: *The authors have declared that no competing interests exist.*  Authors with competing interests  Enter competing interest details beginning with this statement:  *I have read the journal's policy and the authors of this manuscript have the following competing interests: [insert competing interests here]* |  |
| --- | --- |
| * typeset |  |
| Ethics Statement  Enter an ethics statement for this submission. This statement is required if the study involved:   - Human participants - Human specimens or tissue - Vertebrate animals or cephalopods - Vertebrate embryos or tissues - Field research   Write "N/A" if the submission does not require an ethics statement.  General guidance is provided below. Consult the [submission guidelines](http://journals.plos.org/plosone/s/submission-guidelines#loc-guidelines-for-specific-study-types) for detailed instructions. Make sure that all information entered here is included in the Methods section of the manuscript. | LALLF, State Office for Agriculture, Food Safety and Fisheries Mecklenburg-Western Pomerania; LALLF M-V/TSD/7221.3.-1.1-056/12 and 7221.3-1-056/14 |

*Powered by Editorial Manager® and ProduXion Manager® from Aries Systems Corporation*

| Format for specific study types  Human Subject Research (involving human participants and/or tissue)   - Give the name of the institutional review board or ethics committee that approved the study - Include the approval number and/or a statement indicating approval of this research - Indicate the form of consent obtained (written/oral) or the reason that consent was not obtained (e.g. the data were analyzed anonymously)   Animal Research (involving vertebrate animals, embryos or tissues)   - Provide the name of the Institutional Animal Care and Use Committee (IACUC) or other relevant ethics board that reviewed the study protocol, and indicate whether they approved this research or granted a formal waiver of ethical approval - Include an approval number if one was obtained - If the study involved *non-human primates*, add *additional details* about animal welfare and steps taken to ameliorate suffering - If anesthesia, euthanasia, or any kind of animal sacrifice is part of the study, include briefly which substances and/or methods were applied   Field Research  Include the following details if this study involves the collection of plant, animal, or other materials from a natural setting:   - Field permit number - Name of the institution or relevant body that granted permission |  |
| --- | --- |
|  |  |
| Data Availability  Authors are required to make all data underlying the findings described fully available, without restriction, and from the time of publication. PLOS allows rare exceptions to address legal and ethical concerns. See the [PLOS Data Policy](http://journals.plos.org/plosone/s/data-availability) and [FAQ](http://journals.plos.org/plosone/s/data-availability#loc-faqs-for-data-policy) for detailed information. | Yes - all data are fully available without restriction |

*Powered by Editorial Manager® and ProduXion Manager® from Aries Systems Corporation*

| A Data Availability Statement describing where the data can be found is required at submission. Your answers to this question constitute the Data Availability Statement and will be published in the article, if accepted. |  |
| --- | --- |
| Important: Stating ‘data available on request from the author’ is not sufficient. If your data are only available upon request, select ‘No’ for the first question and explain your exceptional situation in the text box. |  |
| Do the authors confirm that all data underlying the findings described in their manuscript are fully available without restriction? |  |
| Describe where the data may be found in full sentences. If you are copying our sample text, replace any instances of XXX with the appropriate details.   - If the data are held or will be held in a public repository, include URLs, accession numbers or DOIs. If this information will only be available after acceptance, indicate this by ticking the box below. For example: *All XXX files are available from the XXX database (accession number(s) XXX, XXX.*). - If the data are all contained within the manuscript and/or Supporting Information files, enter the following: *All relevant data are within the manuscript and its Supporting Information files.* - If neither of these applies but you are able to provide details of access elsewhere, with or without limitations, please do so. For example:   *Data cannot be shared publicly because of [XXX]. Data are available from the XXX Institutional Data Access / Ethics Committee (contact via XXX) for researchers who meet the criteria for access to confidential data.*  *The data underlying the results presented in the study are available from (include the name of the third party* | All relevant data are within the manuscript and its Supporting Information files. |

*Powered by Editorial Manager® and ProduXion Manager® from Aries Systems Corporation*

| *and contact information or URL).*   - This text is appropriate if the data are owned by a third party and authors do not have permission to share the data.   * typeset |  |
| --- | --- |
| Additional data availability information: | Tick here if your circumstances are not covered by the questions above and you need the journal’s help to make your data available. |

*Powered by Editorial Manager® and ProduXion Manager® from Aries Systems Corporation*

<https://www.editorialmanager.com/pone/download.aspx?id=31908186&guid=79562707-d351-4382-bf91-3e760f64fd2a&scheme=1>Manuscript	[Click here to access/download;Manuscript;PONE-D-22-09810 PlosOne manuscript260922.docx](https://www.editorialmanager.com/pone/download.aspx?id=31908186&guid=79562707-d351-4382-bf91-3e760f64fd2a&scheme=1)

1 **Disseminated Intravascular Coagulation (DIC): Old**
2 **player creates new perspectives on the polymicrobial**
3 **sepsis model of CASP.**
4 **Julia van der Linde 1, *, Stephan Diedrich ^1^, Thorben Klee^2^, Claus-Dieter Heidecke^1^,**
5 **Stephan Kersting^1^, Wolfram Keßler^1^**
6 **^1^	Department of General, Visceral, Thoracic and Vascular Surgery,**
7 **University Medical Center Greifswald, Germany**
8 **^2^	Department of Anesthesiology and Intensive Care Medicine, University**
9 **Medical Center Schleswig-Holstein, Lübeck, Germany**

10

11 ***** Correspondence: [Julia.vanderlinde@med.uni-greifswald.de](mailto:Julia.vanderlinde@med.uni-greifswald.de)
12 Department of General, Visceral, Thoracic and Vascular Surgery,
13 University Medical Center Greifswald, Germany

# 14 Abstract:

15 Background: Disseminated Intravascular Coagulation (DIC) is a life-threatening
16 complication of sepsis. In surgical ICUs, DIC is frequently caused by abdominal sepsis,
17 and the disarranged coagulation and complications often lead to death. The severity of
18 sepsis is associated with a higher DIC score according to the parameters proposed by the
19 International Society of Hemostasis and Thrombosis (ISTH) in 2001: platelet count,
20 bleeding time (Quick), D-dimer, and fibrinogen. One problem in studying DIC is finding
21 an adequate animal model that reflects the clinical situation of polymicrobial
22 overwhelming infection.
23 Aims and Methods: We investigated whether a well-established polymicrobial sepsis model
24 of colon ascendens stent peritonitis (CASP) is suited to investigate the complexity of DIC.
25 For this purpose, CASP-operated mice were examined 20 h after the operation with regard
26 to coagulation parameters using cell counts, bleeding times, rotational thromboelastometry
27 (ROTEM), ELISAs for D-dimer and fibrinogen, and platelet accumulation in affected
28 organs via immunohistochemistry to see if the mice develop a coagulation disorder that
29 meets the definition of DIC proposed by the ISTH 2001 consensus conference.
30 Results: Herein, we showed that the CASP model is an all-encompassing animal model to
31 analyze the complexity of systemic DIC in murine abdominal sepsis. There is highly
32 reproducible thrombocytopenia, a significant prolongation of the bleeding time, and a loss
33 of fibrinogen in plasma. We also observed microvascular thrombosis due to platelet
34 accumulation in the microcirculation of the liver.
35 Conclusion: The CASP model seems superior to other artificial models, e.g., injecting
36 substances, for inducing DIC. CASP is one of the best true-to-life models for analyzing
37 the complexity of disseminated intravascular coagulation in polymicrobial sepsis.

38

39 **Keywords:** Sepsis, DIC, Colon Ascendens Stent Peritonitis, Animal Model

# 40 Introduction

41 More than ever, the significance of a misdirected immune response in the context of sepsis
42 with additional disarranged coagulation is relevant. Disseminated intravascular coagulation
43 (DIC), caused by bacterial hyperinflammation, is still not completely understood. New
44 scoring systems and additional criteria to define DIC are currently under evaluation (1, 2).
45 The complexity and necessity of understanding coagulation disorders in the critically ill
46 was highlighted during the COVID-19 pandemic(3), where the influence of coagulation
47 abnormalities on the prognosis of critically ill patients was illustrated.
48 Disseminated intravascular coagulopathy is a life-threatening complication of sepsis. Aside
49 from severe sepsis, other diseases can cause DIC, such as malignancy, trauma, liver
50 cirrhosis, snake bites and obstetric complications. However, sepsis is one of the most
51 common causes of DIC, with DIC being one of the dominant influential factors affecting its
52 outcome (4). The presence of DIC doubles the sepsis mortality rate. During the body’s
53 response to the underlying diseases, there are phases of increased and decreased
54 coagulation characterized by microthrombotic deposits in the capillary flow area or
55 intraparenchymal hemorrhaging in the organ. Thrombotic deposits lead to microinfarction
56 and reduced oxygen supply to the organ, subsequently followed by organ failure with septic
57 shock (5). The pathologic significance of DIC is the simultaneous occurrence of
58 hypercoagulation and hypocoagulation. Developing fibrin deposits increase the
59 consumption of platelets and coagulation factors, and the simultaneous presence of plasmin
60 causes hyperfibrinolysis (6).
61 In 2001, a uniform DIC assessment was drawn up at the consensus conference of the
62 International Society on Thrombosis and Hemostasis (ISTH). The platelet count, bleeding
63 time in the form of the prothrombin index in %, D-dimer level, and fibrinogen level are
64 among the most important parameters of the ISTH score (7). DIC is also often present in
65 sepsis with multiple organ failure.
66 Challenges to developing treatments are its currently unclear basic etiology and the role of
67 its interactions with the immune system. Existing animal DIC models can be categorized
68 according to medication-induced (tissue factor, thrombin or snake poison) and infectious
69 variants, such as DIC caused by LPS (lipopolysaccharide) or bacteria. None of these
70 models present a suitable variant that takes into account the complexity of a polymicrobial
71 infection with a reaction by the immune system. Regarding DIC, the effect of the
72 inflammasome on complex blood components, such as platelets, plays a vital role. Recent
73 data imply that the activation of the inflammasome is the decisive factor in mortality (8).
74 The inflammasome is a multi protein complex belonging to the innate immune system. Triggered by
75 intracellular NOD-like-receptors (NLRs), detecting danger signals in euraryotic cells of
76 bacterial origin, it consists of NLR-protein (a family of 22 NLR), an Adapter protein (ASC)
77 and inflammatory Caspases, e.g. Caspase 1. When activated by
78 intracellular bacterial products e.g. Flagellin or LPS, the inflammatory Caspases lead to Pyrocytosis, a form of lytic
79 cell death, causing the release of Interleukin 1beta and Interleukin 18 (9, 10). In previous
80 studies, our working group was able to demonstrate a strong increase in interleukin 1 beta
81 in the CASP model(11). In Pyrocytosis the plasma membrane rapidly ruptures and
82 thrombogenic substances such as Tissue Factor are released. This pours coals into
83 the fire of DIC and triggers the coagulation cascade(8, 12). Beside this, the inflammasome is
84 currently regarded as misguided immune responses in chronic inflammatory conditions,
85 metabolic syndrome, gout and as a reason for islet cell death in type 2 diabetes in the literature.
87 The current literature criticizes the existing artificial DIC models for not fully incorporating
88 the complexity of sepsis (13). The model of colon ascendens stent peritonitis (CASP) is an intra-abdominal sepsis model
89 created by hollow organ perforation. Pursuant to the S3 consensus conference, human
90 sepsis is defined as a life-threatening situation due to a dysregulated organ response to
91 infection (14). The CASP model seems to reflect the organ dysfunction observed in human
92 sepsis, while other animal models are appropriate to reproduce endotoxin shock (LPS
93 model) or to display systemic inflammatory response syndrome (SIRS), such as cecal
94 ligation and puncture (CLP). In the models involving bacterial pathogens, bacteremia
95 is vital for developing DIC, which is produced in the CASP model by continuous leakage in
96 the area of the cecum.
97 In this paper, we present our comprehensive CASP coagulation analyses. Based on our
98 findings, we recommend using the CASP model for the complex analysis of infectious DIC
99 in animal models because CASP is able to reproducibly recreate derangements of coagulation parameters in the setting of severe polymicrobial
100 sepsis.

# 101 Materials and Methods

102 All experiments were performed on 8- to 10-week-old female C57BL/6 mice. The animals
103 were housed in a temperature-controlled room with a 12-hour light and dark cycle. All
104 experiments were approved by the animal ethics committee of the local animal protection
105 authority (LALLF, State Office for Agriculture, Food Safety and Fisheries Mecklenburg-
106 Western Pomerania; LALLF M-V/TSD/7221.3.-1.1-056/12 and 7221.3-1-056/14). The
107 results shown were created as part of the animal experiments of these projects. In total, the
108 data from 117 C57Bl/6 mice are shown. Different numbers of animals in the groups are
109 originated by our efforts to keep the control groups as small as possible if the experimental
110 setting caused us to expect stable results without a wide range of fluctuation. In addition,
111 we considered that animals in the CASP group could be withdrawn due to excessive
112 stress. In order to avoid small numbers in the CASP group, more animals where included, to
113 obtain meaningful results. All efforts were made to minimize the suffering of the mice,
114 enrichments were provided.

119

## 120 Colon ascendens stent peritonitis (CASP)

121 CASP surgery was carried out according to the method described by Traeger et al. 2010
122 (15). A solution of 0.5 ml xylazine (20 mg/ml), 1.0 ml ketamine (100 mg/ml) and 8.5 ml
123 isotonic NaCl solution was used to anesthetize the mice. By i.p. application of 10 μl
124 anesthetic / g body weight, the animals reached a sufficient depth of anesthesia after about
125 5 minutes. This was checked by the disappearance of pain stimuli on the tail and the
126 inability to trigger leg reflexes. About an hour after application, the animals became lively
127 again and were able to move freely after 2-3 hours.
128 A 16 G cannula (BD Venflon, Franklin Lakes, USA) was introduced 15 mm distal to the
129 ileocecal valve and attached with 7/0 sutures. This stent caused continuous leakage in the
130 area of the cecum and simulated postoperative polymicrobial sepsis. The abdominal wall
131 was closed after repositioning the intestine.

## 132 Analgesia and Monitoring

133 All mice received 0.1 mg/kg buprenorphine intramuscularly per day to treat pain. Stress
134 scores were collected and evaluated every 6 hours. If the stress severity score (SSS) on the
135 animals exceeded a score of 10, they were killed painlessly under anesthesia by cervical
136 dislocation (16). Some values of n in this study are occasionally caused by animals that
137 were exceeded according to their stress severity score (SSS > 10).

## 138 Measurement of the platelet count

139 The platelet count was measured in the whole blood of the model vs. the untreated animals.
140 To do so, we used an impedance-based measurement method (VetScan®, Abaxis, Union
141 City, USA). After 20 hours, the animals’ blood was collected by retro-orbital puncture
142 followed by anticoagulating with EDTA.

## 143 Measurement of the bleeding time

144 To determine the bleeding time, we clipped off 2 mm of the tail tip with a scalpel and
145 measured the time until the onset of hemostasis, following the method of Dejana et al. (17).
146 The bleeding was made visible by transferring the wounded region into a Falcon tube filled
147 with 15 ml of 0.9% NaCl solution at 37 °C. If hemostasis did not occur within 300 sec, the
148 measurement was stopped, and the value was set to 300 sec. The bleeding time was
149 measured in 3 different experiments done within one month, 23 untreated animals were
150 included and 25 CASP-operated animals, we used animals that where proceeded for further
151 analysis, where the Tail-Tip-bleeding test would not interfere with results.

## 152 Rotational thromboelastometry

153 Then, 400 µl of citrate-anticoagulated blood from each mouse was added to ex-Tem® S
154 (tissue thromboplastin) corresponding to the manufacturer’s specifications for determining
155 the extrinsic coagulation cascade as a correlate for testing the prothrombin and clotting time
156 (CT) using ROTEM® delta (TEM international GmbH, Munich, Germany). The
157 ascertained values were plotted against each other. Because the analyzer can only evaluate
158 4 samples at the same time, data from two experimental days were collected to minimize
159 the time between blood sampling and analysis of the probes in ROTEM® delta.

160

## 161 ELISA

### 162 D-dimer ELISA

163 To analyze the D-dimer concentration of the septic and untreated mice, plasma at a 1:6
164 dilution was measured with the *Stago* Asserachrom® D-dimer ELISA kit **(**Asnieres sur
165 Seine, France). All steps were carried out according to the manufacturer’s specifications,
166 measuring the OD after terminating the reaction on the ELISA reader at 450 nm. Standard
167 curves and concentrations were prepared and analyzed using GraphPad Prism 8 software.

### 168 Fibrinogen ELISA

169 The enzyme-linked immunosorbent assay (ELISA, by the company *Abnova,*Taipei,
170 Taiwan) was conducted with an anti-fibrinogen antibody precoated 96-well microtiter plate
171 according to the manufacturer’s specifications. The plate was incubated with diluted mouse
172 plasma (1:30,000) for 10 minutes at room temperature. The plasma was produced from
173 anticoagulated whole mouse blood by centrifugation for 15 minutes at 2,000 g. Reaction
174 detergents were added according to the ELISA manufacturer’s specifications. The optical
175 density was measured using an ELISA microplate reader after terminating the reaction.
176 Standard curves and concentrations were prepared and analyzed using GraphPad Prism 8
177 software.

## 178 Immunohistological staining

179 The organ cryosections were each incubated with 20 µl 20% FCS buffer for 10 minutes at
180 room temperature and then treated with peroxidase blocking reagent for 10 minutes,
181 followed by washing the sections twice with PBS. For single staining of platelets in the
182 sections of the liver, anti-fibrinogen antibodies were not applied. The primary antibody
183 anti-CD42c FITC (Emfret, Würzburg, Germany) was incubated at 5 µl in 100 µl 20% FCS
184 buffer at 4 °C, the sections were washed again, and then incubated with the secondary
185 antibody Alexa 488 at a dilution of 1:100.
186 We used 100 µl of the antibody mixture for the double staining of fibrinogen/CD42c
187 (CD42c 1:100; fibrinogen 1:50 in PBS/20% FCS), which was incubated at 4 °C. Serving as
188 an isotype control, the second organ section was treated with PBS/20% FCS (the isotype
189 controls are not shown). Subsequently, the sections were incubated with 50 µl of the
190 peroxidase-coupled secondary antibody (1:100). After additional washing, the organ
191 sections were immersed in amplification diluent with 50 µl biotonyl tyramide (1:200), and
192 after washing, 50 µl of anti-FITC-Alexa 488 (1:100) and streptavidin TRITC (1:200) were
193 added in PBS/20% FCS. In the final step, the organ sections were treated with fluoroprep,
194 and after examination with a Keyence Fluorescence Microscope BZ-9000 (Keyence,
195 Osaka, Japan) at 20 x optic, all microscope images were evaluated using the BZ-II
196 Analyzer. The TRITC positive area was analyzed with ImageJ software.

## 197 Hematoxylin-eosin (H.E.) staining

198 The liver removed postmortem was embedded in paraffin-blocks, according to a standard
199 protocol, sectioned into 3 µm slices on the microtome, fixed on a slide and stained. Sections
200 were deparaffinized in two consecutive xylene baths for 5 min each. The sections were
201 hydrated using a descending ethanol series (95%, 80%, 70%; 5 min each) and rinsed in
202 double-distilled water. This was followed by incubation in Mayer's hematoxylin solution for
203 15 minutes before rinsing again with double distilled water. Subsequent counterstaining was
204 performed with eosin Y and acetic acid for 4 min followed by rinsing twice. Dehydration
205 was then carried out using an ascending ethanol series (70%, 80%, 90%) and cleaning with
206 two xylene baths for 5 min each. The sections were covered with glycerine gelatin.

## 207 Statistical analysis

208 Statistical analysis was calculated with GraphPad Prism version 8.0.0 for Windows,
209 GraphPad Software, San Diego, California USA. Data are presented as medians and interquartile ranges (IQR). Differences between the median values was tested using a Mann–Whitney test . Statistical significance was accepted at a two-tailed p-value of <0.05.

# 210 Results

## 211 Platelet count

212 The platelet count in the septic animals dropped very significantly to values well below the
213 normal range (Figure 1). The experiments were carried out repeatedly, and all mice
214 developed thrombocytopenia 20 hours after CASP induction. We were able to show a
215 significant decrease in the platelet count to 370.000 platelets/µl below the reference value
216 of mouse C57Bl/6 (450.000–900.000 platelets/µl), reproducibly occurred 20 hours after
217 inducing sepsis. Counts below 450.000 platelets/µl were consistently reproducible after
218 CASP surgery, decreasing from 7.1 (1.76 IQR interquartile range IQR) × 10^9^ platelets/ml to
219 3.14 (3.46 IQR) × 10^9^ platelets/ml. On average, the model mice lost approximately 60% of
220 their circulating platelets (p = 0.0016; n = 5 untreated group; n = 8 CASP group).

###### 221 Figure 1

222 Fig 1. Platelet count 20 hours after CASP surgery
223 Untreated mice had a mean platelet count of 7.1 (1.76 IQR) × 10^9^/ml, white column. Mice that
224 underwent CASP surgery showed a reduction to 3.14 (3.46 IQR) × 10^9^/ml, black column. Septic mice lost
225 60% of their platelets. The decrease in platelet count was highly significant (p = 0,0016**; n = 5 untreated
226 group; n = 8 CASP group). The bold black line depicts the median.

## 227 Tail tip bleeding time

228 We analyzed the bleeding time of septic and untreated mouse tail tips. Septic mice showed
229 a significantly prolonged bleeding time compared to the untreated control animals (Figure
230 2). On average, the median bleeding time of septic animal tail tips was 93 (260 IQR)
231 seconds. In contrast, untreated animals showed a bleeding time of 57 (197 IQR) seconds.
232 This difference was highly significant (p < 0.001; n = 23 untreated group; n = 25 CASP
233 group).

###### 234 Figure 2

235 Fig 2 Tail tip bleeding time in seconds
236 Shown are bleeding times out of the tail tip of 8- to 10-week-old C57Bl/6 mice divided into CASP-
237 operated, black column, and untreated animals, white column, 20 hours after CASP induction exhibiting
238 significant differences in bleeding time (p < 0.001***; n = 23 untreated group; n = 25 CASP group). The tail
239 tip bleeding time was 93 (260 IQR) seconds in septic mice and untreated mice showed a bleeding time of 57
240 (197 IQR) seconds. The bold black line depicts the median.

## 241 Thromboelastometry

### 242 Clotting time (CT) – enzyme activity

243 Rotational thromboelastometry (ROTEM) was used to obtain a correlate of the prothrombin
244 time test according to the specifications of the ISTH 2001 DIC criteria. Clotting time (CT)
245 was determined using EXTEM. Clot formation time and MCF were also measured, but data
246 are not shown due to their similar results to the CT. After 20 hours of CASP induction, we
247 detected a prolonged median CT in the model mice of 109 (409 IQR) seconds, while the
248 untreated control animals showed a CT of 80 (21 IQR) seconds. The variances were
249 significantly different. The results are depicted in Figure 3. This difference also proved to
250 be highly reproducible and the p value was p = 0.0043 (n = 6 untreated group; n = 5 CASP
251 group).

###### 252 Figure 3

253 Fig 3 Clotting time (CT) extrinsic pathway rotational thromboelastometry ROTEM I
254 Illustrated is the CT, measured via rotational thromboelastometry EXTEM in seconds of female 8- to 10-
255 week-old C57Bl/6 mice untreated (white column) or 20 h after CASP surgery (CASP, black column); control
256 mice (untreated) exhibited a stable clotting mechanism within 80 (21 IQR) seconds while 20 h after CASP
257 surgery, the mice showed a prolongation of CT to 109 (409 IQR) seconds (p = 0.0043**; n = 6 untreated
258 group; n = 5 CASP group). The bold black line depicts the median.

### 259 D-dimer

260 D-dimer levels were also calculated using ELISA. Not surprisingly, 20 h after CASP
261 surgery, there was a significant increase in the blood levels of D-dimer (Fig. 4). The basal
262 D-dimer levels of the untreated animals showed median values of approximately 120.9
263 (66.2 IQR) ng/ml, while 20 h after CASP surgery, it increased to 278.6 (722.9) ng/ml (p <
264 0.0001; n = 8 untreated group; n = 11 CASP group).

###### 265 Figure 4

266 Fig 4*.* D-dimer levels in the blood of untreated or CASP-operated C57Bl/6 mice.
267 Statistical results of D-dimer levels 20 h after CASP surgery (CASP, black column) or untreated (white
268 column) 8- to 10-week-old C57Bl/6. Basal blood levels of untreated animals 120.9 (66.2 IQR) ng/ml
269 increased significantly to 278.6 (722.9) ng/ml in CASP-operated mice (p < 0.0001****; n = 8 untreated
270 group; n = 11 CASP group); The bold black line depicts the median.

### 271 Fibrinogen

272 Fibrinogen analysis in the plasma of septic mice was carried out by comparing septic with
273 untreated mice using a sandwich ELISA technique, which revealed a significant decrease
274 (CASP: 29.71 (126.3 IQR) mg/dl blood plasma vs. untreated: 185.2 (83.91 IQR) mg/dl
275 blood plasma) (p = 0.03, n = 4 untreated group, n = 5 CASP group). The results are
276 depicted in Figure 5. These results could be verified by morphological imaging via
277 immunohistochemical staining (Figure 6).

###### 278 Figure 5

279 Fig. 5 Fibrinogen blood levels 20 h after CASP surgery
280 Statistical results of fibrinogen blood levels 20 h after CASP surgery in 8- to 10-week-old C57BL/6
281 mice depicting a significant decrease in fibrinogen plasma levels in CASP-operated mice (CASP, black
282 column). Control animals (untreated, white column) showed 185.2 (83.91 IQR) mg/dl vs. 29.71 (126.3 IQR)
283 mg/dl blood levels 20 h after CASP surgery (p = 0.03*; n = 4 untreated group; n = 5 CASP group). The bold
284 black line depicts the median.

### 285 Platelet deposits in the liver

286 To demonstrate thrombus formation in organs, we conducted H.E. staining of the liver, spleen and lung. but found evidence of thrombus formation only in the liver. To answer the question
287 regarding the fate of the missing platelets, we proceeded with further platelet-
288 specific staining of the respective organs and found significant platelet accumulation in the
289 microvascular bed of the liver, as shown in Figure 7. Here, we present representative results
290 and summarize the results in Figure 8. We could clearly demonstrate a significant increase
291 in the accumulation of platelets in the liver.
292 Compared to the untreated control group (p = 0.0002), the liver of C57Bl/6 mice showed a
293 significant increase in the platelet-specific marker CD42c (green dye Figure 8). On average,
294 the control mice had a FITC-positive area of 0.54% (0.6% IQR) (n = 10). The medium
295 fluorescence signal in the CASP-operated animals increased to 1.77% (1.69% IQR) of the
296 area (n = 7). We could not find any differences in spleen and lung.

###### 297 Figure 6

Fig 6. Frozen sections of the liver with immunohistochemistry staining with an anti-fibrinogen antibody Alexa 488 and platelet anti-CD 42c antibody TRITC. Pictured: left row untreated control animals (a-c) showing liver sections of untreated 8- to 10-week-old C57Bl/6 (3 examples a-c untreated and d-f CASP), the green dye (Alexa 488) represents the fibrinogen amount in the liver sinusoids, the orange dye (TRITC) shows platelet aggregates, the right column is 20 h after CASP surgery (d-f, scale bar 100 µm, negative control not shown).

298 The double staining of fibrinogen and platelets in CASP sepsis clearly verified increased
299 fibrinogen in the area of the sinusoids in untreated mice, while fibrinogen was less readily
300 seen in those with sepsis.

###### 301 Figure 7

Fig 7. Immunohistochemical staining of frozen liver sections 20 h after CASP with anti-CD42c*FITC/Alexa 488. Depicted are representative results of frozen sections from 8- to 10-week-old C57Bl/6 liver tissue with immunohistochemistry staining of platelets (green dye) with anti-CD 42c antibody*FITC/Alexa 488. The left row shows only a few platelets uniformly distributed in the liver tissue of untreated animals (a), while the right row shows platelet accumulation and aggregates in the liver tissue 20 h after CASP surgery (b); (scale bar 100 µm, negative control not shown, quantification

###### 302 Figure 8

303 Fig 8. Accumulation of hepatic platelets 20 hours after CASP surgery.
304 Pictured is the percentage area of FITC/Alexa 488-positive areas on the fluorescent section in proportion to
305 the entire section. There was a significant increase (p < 0.001***) in the percentage of the FITC/Alexa 488-
306 positive area, i.e., an increased accumulation of hepatic platelets in CASP-operated animals of 1.77% (1.69%
307 IQR) of the area (n = 7) compared to untreated animals with 0.54% (0.6% IQR) of the FITC-positive area (n =
308 10). The bold black line depicts the median.

# 309 Discussion

310 DIC is a very complex and dynamic process, and coagulation tests show a point-in-time snapshot of the coagulation situation, with coexisting
311 increased coagulation and hyperfibrinolysis. To understand the complexity of this process,
312 it is reasonable to transfer the ISTH 2001 criteria to the murine system for DIC research
313 (13). Admittedly, there are other criteria that are well known, e.g., the Japanese Ministry of
314 Health and Welfare (JMHW) criteria and the Japanese Association for Acute Medicine
315 (JAAM) criteria, each of which has advantages and disadvantages. We applied the ISTH
316 criteria because they are well established (2, 18) and were previously used in other animal
317 studies(13).
318 Differences from the human progression of sepsis have been observed in animal models.
319 The fulminant sepsis of CASP-operated mice triggers hyperinflammation followed by
320 immunosuppression. In the human system, however, the phase of hyperinflammation is
321 often absent, and there is immunosuppression or hypoinflammation instead (19). The
322 different initial immunological situations of mice and humans may also produce variations
323 in the frequency of thrombocytopenia. Only approximately 54% of human septic cases
324 develop thrombocytopenia (20). In our current paper, however, almost all CASP-operated
325 mice developed a significantly lower platelet count after 20 hours (see Fig. 1). Hence, the first vital aspect of the ISTH score,
326 thrombocytopenia, could be met in CASP. Clinical studies have shown that the extent of
327 thrombocytopenia correlates with a poor survival rate in critically ill patients, thus being a
328 negative predicative factor (23). Thrombocytopenia (below 150,000 Plt/µl) in the setting of sepsis may develop
329 in humans through the involvement of platelets in the immune response to Gram-positive
330 and Gram-negative bacteria. Platelets, in their role as covercytes, can directly interact with
331 and eliminate bacteria, causing them to be removed from circulation (24). An additional reason for
332 the platelet decline may be the accumulation of platelets in the liver and spleen (25). Severe
333 sepsis may result in the already mentioned DIC and, by developing microthrombi in the
334 atrial flow area, lead to multiple organ failure.
335 Due to thrombocytopenia being detected in the murine model, we were also interested
336 in the question of whether thrombocytopenia influenced coagulation times. Owing to the
337 fact that a healthy mouse has many more platelets in its circulation than a healthy human, it
338 was questionable whether thrombocytopenia below 450,000 Plt/µl even had any influence
339 on murine coagulation. This was, however, definitively answered by the prolonged
340 bleeding time of the septic animals compared to the untreated control animals (see Fig. 2).
341 An additional parameter for examining coagulation function is the mentioned
342 prothrombin index in %, also referred to as the “Quick”-value. This value examines the
343 extrinsic coagulation cascade. We analyzed the extrinsic coagulation cascade as an
344 equivalent using rotational thromboelastometry (ROTEM®). To measure the coagulation
345 time, we used thromboplastin with this test kit (tissue factor; ex-Tem®). As a result, we
346 were able to show that the CASP-operated mouse had a longer extrinsic coagulation time
347 (Fig. 3). Admittedly, our data show the results of small groups (n= 6 untreated group; n = 5
348 CASP group) and present a large IQR of 409 s in the CASP-operated group, which is
349 comparatively high relative to the IQR of 21 s in the untreated group. An explanation for
350 the small group sizes is that the ROTEM®-Analyzer can test 4 probes in parallel, but since
351 time is an important factor in analyzing blood clotting, we minimized the time from taking
352 blood samples to analysis; therefore, 2 experimental cycles were performed, and the tests
353 were performed immediately after blood extraction. Due to the septic circulation conditions
354 of the animals, it was not possible to obtain enough blood for multiple samples from any
355 CASP-operated mouse.
356 In hospitals, thromboelastometry is an appropriate tool to rapidly obtain an overview of
357 the performance of patients’ intrinsic and extrinsic coagulation parameters. This method is
358 of particular relevance in surgical transplantation and sepsis therapy (26). Researchers were
359 able to show that the 30-day survival probability was 85.7% among patients with normal
360 ROTEM® parameters. If there was only one change in the measured coagulation
361 parameters, the 30-day survival probability declined to 58.7% (21). This observation is a
362 strong indication of a lack of coagulation factors. Furthermore, additional criteria of the
363 ISTH Consensus Conference 2001 could also be confirmed in our model. There were
364 elevated D-dimer levels as well as decreased fibrinogen levels in the septic animals.
365 With regard to the severe thrombocytopenia in CASP, the question then was what had
366 happened to the platelets missing from the circulation. To answer this question, we
367 prepared frozen immunohistological sections of the liver, lung, and spleen. Our study was
368 able to show that the liver parenchyma developed significant platelet accumulation 20
369 hours after CASP surgery (Fig. 6a-f and 7a, b). In doing so, the septic animals showed
370 rather diffusely distributed platelet aggregations (Fig. 6d-f TRITC orange dye, 7b Alexa
371 488 green dye), while the untreated animals displayed regularly distributed, individual
372 platelets in the parenchyma (Fig. 6a-c, 7a). There were, however, fewer platelets in the area
373 of central veins (Fig. 7b) in septic animals than in untreated control animals. An increase in
374 the pooling of platelets in the liver of septic animals could also be verified by a significant
375 increase in the fluorescent intensity after staining the platelets with CD42c*FITC/Alexa488
376 (Figure 7a, b). The pathological mechanisms of platelet accumulation are the subject of
377 comprehensive analyses in the current literature. Aside from forming agglomerations, there
378 are other explanations for their increased accumulation in the liver tissue. The literature
379 also describes examinations of other models, such as the cecal ligation and puncture (CLP)
380 and the LPS shock model, showing that platelets induce, among other effects, the
381 recruitment of neutrophils into the vascular bed of the liver where they develop aggregates
382 (27). The sinusoids, situated behind these platelet-leukocyte aggregates, are then
383 underperfused. The platelets also add to septic liver cell damage through interactions with
384 neutrophil granulocytes (28). A crucial factor for the accumulation of platelets in the
385 capillary bed of the liver is the thrombocytic adhesion molecule CD62p. Other authors
386 describe the accumulation of platelets as the body’s defense mechanism against increased
387 coagulopathy in the setting of DIC (29). The hepatocyte asialoglycoprotein receptor, also
388 referred to as the Ashwell receptor, was discovered in 2008 (30). It is able to sequester
389 platelets in the setting of a Gram-negative, systemic infection. For this purpose,
390 bacterial neuraminidases help sequester the n-terminal acetyl lactosamine acids of
391 glycoproteins on the host cells to allow their use in their own energy balance (31). Bacteria
392 that have neuraminidases in their repertoire of virulence factors and belong to the typical
393 intestinal flora are, for example, *Bacteroides* spp. and *Escherichia coli*. Hence, the Ashwell
394 receptor might also produce platelet aggregation in the liver of CASP models. However, the
395 exact aggregation pathomechanism was not examined in this research paper.
396 The histological examinations could not identify a significantly increased aggregation
397 of platelets in the spleen. Human autopsies, too, only inconsistently reveal significant
398 microthrombosis in patients with a high DIC score (32, 33). The reason for the difficult
399 histological detection of microthrombosis might be the fact that DIC simultaneously causes
400 hyperfibrinolysis, which might have already removed the thromboses that occurred. The
401 chosen method of immunohistochemistry might not have been appropriate for our model to
402 illustrate sequestered platelets in the spleen. It might be that platelet sequestration by
403 splenic macrophages triggered a reaction in the platelets, causing loss of the CD42c antigen.
405 In sepsis, neutrophil platelet aggregates may develop in the capillary flow area of
406 the lung (34). The aggregation of platelets in lungs during abdominal sepsis in humans as
407 well as peritonitis in mice is well documented in the literature (35, 36). The
408 immunohistochemical analyses showed striking variation in the platelet aggregations in the
409 alveolar flow area, and were thus inconconclusive.
410 We were able to reconstruct DIC clinical parameters that met the definition of the
411 ISTH 2001 Consensus Conference (7) in the CASP model. There was a significant decrease
412 in the platelet count, the plasma fibrinogen level was significantly reduced, and the
413 bleeding time was significantly prolonged. We were also able to show that this led to
414 platelet aggregation in the liver parenchyma. D-dimer is an additional parameter of DIC.
415 This fibrin degradation product gives information on plasmin activity and fibrinolysis. A
416 study by the University of Zurich showed that an increase in D-dimer above the normal
417 reference value occurred after abdominal surgical interventions. The maximum value was
418 reached on the seventh postoperative day, with the values returning to the reference range
419 only on the twenty-fifth postoperative day (37). Since CASP is a surgical model for
420 inducing severe sepsis, an elevation of D-dimer can be expected 20 hours after CASP
421 surgery. Consequently, the elevated D-dimer level had no significance for assessing DIC in
422 the model used.
423 Thus, we have been able to verify all ISTH 2001 parameters that define DIC, and from
424 our point of view, the CASP model is an appropriate animal model to exemplify sepsis-
425 induced DIC. As a result, DIC can be studied in this complex context, such as in terms of
426 its medication responses, in a highly reproducible animal model.

## 427 Conclusion

428 The CASP model is appropriate to analyze DIC diagnostic criteria. Currently, there are
429 few appropriate DIC animal models with the majority being created by injecting
430 substances, such as thromboplastin or lipopolysaccharide, to generate increased coagulation
431 (13). These models lack realistic simulation of severe sepsis. The advantage of the CASP
432 model is its more realistic simulation of polymicrobial sepsis following a major abdominal
433 surgical intervention. Additionally, the model provides the opportunity to analyze the effect
434 of new medications on septic animals and to detect the influence of coagulopathy on
435 outcomes. All in all, the CASP model is suitable to analyze the complexity of DIC in
436 polymicrobial sepsis.

###### 437 Author Contributions:

438 Conceptualization and project design: JvdL; methodology and performance of
439 experiments: JvdL, TK; data evaluation: JvdL, TK; interpretation of data: JvdL, SD, WK,
440 CDH, SK; writing - original draft preparation: Jvdl, SK, SD, CDH and WK; writing -
441 review and editing: all authors critically reviewed the manuscript.
442 **Funding:** This work was financially supported by the German Research Foundation
443 (RTG 840; grants to JvdL). The funders had no role in study design, data collection and
444 analysis, decision to publish, or preparation of the manuscript.
445 **Acknowledgments:** We would like to thank the Institute of Immunology and
446 Transfusion Medicine, Department of Transfusion Medicine, University Medicine
447 Greifswald for their assistance and opportunity to work with ROTEM® delta. Especially, I
448 would like to express my gratitude to and respect for Birgitt Fürll. She was an inspiring and
449 extraordinary human and a dedicated scientist, with a passion for platelets; unfortunately,
450 she passed away.
451 **Conflicts of Interest:** The authors declare that they have no known competing
452 financial interests or personal relationships that could have appeared to influence the work
453 reported in this paper.
454 **References**
455 1.	Iba T, Connors JM, Nagaoka I, Levy JH. Recent advances in the research and management of sepsis-associated DIC.
456 Int J Hematol. 2021;113(1):24-33.
457 2.	Levi M. Another step in improving the diagnosis of disseminated intravascular coagulation in sepsis. Critical care
458 (London, England). 2013;17(4):448-.
459 3.	Tang N, Li D, Wang X, Sun Z. Abnormal coagulation parameters are associated with poor prognosis in patients with
460 novel coronavirus pneumonia. J Thromb Haemost. 2020;18(4):844-7.
461 4.	Akca S, Haji-Michael P, de Mendonça A, Suter P, Levi M, Vincent JL. Time course of platelet counts in critically ill
462 patients. Crit Care Med. 2002;30(4):753-6.
463 5.	Rice TW, Wheeler AP. Coagulopathy in critically ill patients: part 1: platelet disorders. Chest. 2009;136(6):1622-30.
464 6.	Samuels JM, Moore HB, Moore EE. Coagulopathy in Severe Sepsis: Interconnectivity of Coagulation and the Immune
465 System. Surg Infect (Larchmt). 2018;19(2):208-15.
466 7.	Taylor FB, Jr., Toh CH, Hoots WK, Wada H, Levi M. Towards definition, clinical and laboratory criteria, and a
467 scoring system for disseminated intravascular coagulation. Thromb Haemost. 2001;86(5):1327-30.
468 8.	Wu C, Lu W, Zhang Y, Zhang G, Shi X, Hisada Y, et al. Inflammasome Activation Triggers Blood Clotting and Host
469 Death through Pyroptosis. Immunity. 2019;50(6):1401-11.e4.
470 9.	Martinon F, Burns K, Tschopp J. The Inflammasome: A Molecular Platform Triggering Activation of Inflammatory
471 Caspases and Processing of proIL-&#x3b2. Molecular Cell. 2002;10(2):417-26.
472 10. Dagenais M, Skeldon A, Saleh M. The inflammasome: in memory of Dr. Jurg Tschopp. Cell Death Differ.
473 2012;19(1):5-12.
474 11. Maier S, Traeger T, Entleutner M, Westerholt A, Kleist B, Hüser N, et al. Cecal ligation and puncture versus colon
475 ascendens stent peritonitis: two distinct animal models for polymicrobial sepsis. Shock. 2004;21(6):505-11.
476 12. Bergsbaken T, Fink SL, Cookson BT. Pyroptosis: host cell death and inflammation. Nat Rev Microbiol. 2009;7(2):99-
477 109.
478 13. Berthelsen LO, Kristensen AT, Tranholm M. Animal models of DIC and their relevance to human DIC: A systematic
479 review. Thrombosis Research. 2011;128(2):103-16.
480 14. Singer M, Deutschman CS, Seymour CW, Shankar-Hari M, Annane D, Bauer M, et al. The Third International
481 Consensus Definitions for Sepsis and Septic Shock (Sepsis-3). JAMA. 2016;315(8):801-10.
482 15. Au - Traeger T, Au - Koerner P, Au - Kessler W, Au - Cziupka K, Au - Diedrich S, Au - Busemann A, et al. Colon
483 Ascendens Stent Peritonitis (CASP) - a Standardized Model for Polymicrobial Abdominal Sepsis. JoVE. 2010(46):e2299.
484 16. Kiank C, Holtfreter B, Starke A, Mundt A, Wilke C, Schütt C. Stress susceptibility predicts the severity of immune
485 depression and the failure to combat bacterial infections in chronically stressed mice. Brain Behav Immun. 2006;20(4):359-
486 68.
487 17. Dejana E, Callioni A, Quintana A, de Gaetano G. Bleeding time in laboratory animals. II - A comparison of different
488 assay conditions in rats. Thrombosis Research. 1979;15(1):191-7.
489 18. Kobayashi N, Maekawa T, Takada M, Tanaka H, Gonmori H. Criteria for diagnosis of DIC based on the analysis of
490 clinical and laboratory findings in 345 DIC patients collected by the Research Committee on DIC in Japan. Bibl Haematol.
491 1983(49):265-75.
492 19. Koerner P, Westerholt A, Kessler W, Traeger T, Maier S, Heidecke CD. Surgical trauma and postoperative
493 immunosuppression. Chirurg. 2008;79(4):290-+.
494 20. Tsirigotis P, Chondropoulos S, Frantzeskaki F, Stamouli M, Gkirkas K, Bartzeliotou A, et al. Thrombocytopenia in
495 critically ill patients with severe sepsis/septic shock: Prognostic value and association with a distinct serum cytokine profile.
496 Journal of Critical Care. 2016;32:9-15.
497 21. Adamzik M, Langemeier T, Frey UH, Gorlinger K, Saner F, Eggebrecht H, et al. COMPARISON OF
498 THROMBELASTOMETRY WITH SIMPLIFIED ACUTE PHYSIOLOGY SCORE II AND SEQUENTIAL ORGAN
499 FAILURE ASSESSMENT SCORES FOR THE PREDICTION OF 30-DAY SURVIVAL: A COHORT STUDY. Shock.
500 2011;35(4):339-42.
501 22. Traeger T, Koerner P, Kessler W, Cziupka K, Diedrich S, Busemann A, et al. Colon ascendens stent peritonitis
502 (CASP)--a standardized model for polymicrobial abdominal sepsis. J Vis Exp. 2010(46):2299.
503 23. Crowther MA, Cook DJ, Meade MO, Griffith LE, Guyatt GH, Arnold DM, et al. Thrombocytopenia in medical-
504 surgical critically ill patients: prevalence, incidence, and risk factors. J Crit Care. 2005;20(4):348-53.
505 24. White JG. Platelets are covercytes, not phagocytes: uptake of bacteria involves channels of the open canalicular
506 system. Platelets. 2005;16(2):121-31.
507 25. Itoh H, Cicala C, Douglas GJ, Page CP. Platelet Accumulation Induced By Bacterial Endotoxin In Rats. Thrombosis
508 Research. 1996;83(6):405-19.
509 26. Sivula M, Pettila V, Niemi TT, Varpula M, Kuitunen AH. Thromboelastometry in patients with severe sepsis and
510 disseminated intravascular coagulation. Blood Coagulation & Fibrinolysis. 2009;20(6):419-26.
511 27. Singer G, Urakami H, Specian RD, Stokes KY, Granger DN. Platelet recruitment in the murine hepatic
512 microvasculature during experimental sepsis: role of neutrophils. Microcirculation. 2006;13(2):89-97.
513 28. Yadav SS, Howell DN, Steeber DA, Harland RC, Tedder TF, Clavien PA. P-Selectin mediates reperfusion injury
514 through neutrophil and platelet sequestration in the warm ischemic mouse liver. Hepatology. 1999;29(5):1494-502.
515 29. Grozovsky R, Hoffmeister KM, Falet H. Novel clearance mechanisms of platelets. Curr Opin Hematol.
516 2010;17(6):585-9.
517 30. Grewal PK, Uchiyama S, Ditto D, Varki N, Le DT, Nizet V, et al. The Ashwell receptor mitigates the lethal
518 coagulopathy of sepsis. Nat Med. 2008;14(6):648-55.
519 31. Grewal PK. THE ASHWELL-MORELL RECEPTOR. Methods in Enzymology, Vol 479: Functional Glycomics.
520 Methods in Enzymology. 479. San Diego: Elsevier Academic Press Inc; 2010. p. 223-41.
521 32. Watanabe T, Imamura T, Nakagaki K, Tanaka K. Disseminated Intravascular Coagulation in Autopsy Cases Its
522 Incidence and Clinicopathologic Significance. Pathology - Research and Practice. 1979;165(4):311-22.
523 33. Rizoli S, Nascimento BJ, Key N, Tien HC, Muraca S, Pinto R, et al. Disseminated Intravascular Coagulopathy in the
524 First 24 Hours After Trauma: The Association Between ISTH Score and Anatomopathologic Evidence. The Journal of
525 Trauma and Acute Care Surgery. 2011;71(5):S441-S7 10.1097/TA.0b013e318232e688.
526 34. Welbourn CRB, Young Y. Endotoxin, Septic Shock and acute Lung Injury - Neutrophils, Macrophages and
527 Inflammatory Mediators 1992.
528 35. Asaduzzaman M, Lavasani S, Rahman M, Zhang S, Braun OO, Jeppsson B, et al. Platelets support pulmonary
529 recruitment of neutrophils in abdominal sepsis. Crit Care Med. 2009;37(4):1389-96.
530 36. Kornerup KN, Salmon GP, Pitchford SC, Liu WL, Page CP. Circulating platelet-neutrophil complexes are important
531 for subsequent neutrophil activation and migration. Journal of Applied Physiology. 2010;109(3):758-67.
532 37. Dindo Da, Breitenstein Sa, Hahnloser Da, Seifert Bb, Yakarisik Sa, Asmis LMc, et al. Kinetics of D-dimer after
533 general surgery. Blood Coagulation & Fibrinolysis July. 2009;20(5):347-52.

546

547

548 Supporting Information
549 S1 Appendix
550 S 1. None accumulation of platelets in spleen and lung 20 hours after CASP surgery.
551 Immunohistochemical staining of frozen spleen and lung sections 20 h after CASP with anti-
552 CD42c*FITC/Alexa 488. Depicted are representative results of frozen sections from 8- to 10-week-old
553 C57Bl/6 organ tissue with immunohistochemistry staining of platelets with anti-CD 42c
554 antibody*FITC/Alexa 488. The upper section shows distribution of platelets in spleen tissue in untreated mice
555 (S1a) and CASP operated (S1b), while the lower section shows platelet dye in the lung tissue untreated (S1c)
556 and 20 h after CASP surgery (S1d); (scale bar in µm).

Supporting Information - Compressed/ZIP File Archive

#### [Click here to access/download](https://www.editorialmanager.com/pone/download.aspx?id=31908290&guid=2e4eaaf0-6a35-4e44-9060-fa77d18119dd&scheme=1)

[Supporting Information - Compressed/ZIP File Archive PLOS_20220420_41511PM.zip](https://www.editorialmanager.com/pone/download.aspx?id=31908290&guid=2e4eaaf0-6a35-4e44-9060-fa77d18119dd&scheme=1)

Revised Manuscript with Track Changes

1 **Disseminated Intravascular Coagulation (DIC): Old**
2 **player creates new perspectives on the polymicrobial**
3 **sepsis model of CASP.**
4 **Julia van der Linde 1, *, Stephan Diedrich ^1^, Thorben Klee^2^, Claus-Dieter Heidecke^1^,**
5 **Stephan Kersting^1^, Wolfram Keßler^1^**
6 **^1^	Department of General, Visceral, Thoracic and Vascular Surgery,**
7 **University Medical Center Greifswald, Germany**
8 **^2^	Department of Anesthesiology and Intensive Care Medicine, University**
9 **Medical Center Schleswig-Holstein, Lübeck, Germany**

10

11 ***** Correspondence: [Julia.vanderlinde@med.uni-greifswald.de](mailto:Julia.vanderlinde@med.uni-greifswald.de)
12 Department of General, Visceral, Thoracic and Vascular Surgery,
13 University Medical Center Greifswald, Germany

##### 14 Abstract:

15 Background: Disseminated Intravascular Coagulation (DIC) is a life-threatening
16 complication of sepsis. In surgical ICUs, DIC is ~~mainly~~ frequently caused by abdominal
17 sepsis, and the disarranged coagulation and complications often lead to death. The severity
18 of sepsis is associated with a higher DIC score according to the parameters proposed by
19 the International Society of Hemostasis and Thrombosis (ISTH) in 2001: platelet count,
20 bleeding time (Quick), D-dimer, and fibrinogen. One problem in studying DIC is finding
21 an adequate animal model that reflects the clinical situation of polymicrobial
22 overwhelming infection.
23 Aims and Methods: We investigated whether a well-established polymicrobial sepsis model
24 of colon ascendens stent peritonitis (CASP) is suited to investigate the complexity of DIC.
25 For this purpose, CASP-operated mice were examined 20 h after the operation with regard
26 to coagulation parameters using cell counts, bleeding times, rotational thromboelastometry
27 (ROTEM), ELISAs for D-dimer and fibrinogen, and platelet accumulation in affected
28 organs via immunohistochemistry to see if the mice develop a coagulation disorder that
29 meets the definition of DIC proposed by the ISTH 2001 consensus conference.
30 Results: Herein, we showed that the CASP model is an all-encompassing animal model to
31 analyze the complexity of systemic DIC in murine abdominal sepsis. There is highly
32 reproducible thrombocytopenia, a significant prolongation of the bleeding time, and a loss
33 of fibrinogen in plasma. We also observed microvascular thrombosis due to platelet
34 accumulation in the microcirculation of the liver.
35 Conclusion: The CASP model seems superior to other artificial models, e.g., injecting
36 substances, for inducing DIC. CASP is one of the best true-to-life models for analyzing
37 the complexity of disseminated intravascular coagulation in polymicrobial sepsis.

38

39 **Keywords:** Sepsis, DIC, Colon Ascendens Stent Peritonitis, Animal Model

##### 40 Introduction

41 More than ever, the significance of a misdirected immune response in the context of sepsis
42 with additional disarranged coagulation is relevant. Disseminated intravascular coagulation
43 (DIC), caused by bacterial hyperinflammation, is still not completely understood. New
44 scoring systems and additional criteria to define DIC are currently under evaluation (1, 2).
45 The complexity and necessity of understanding coagulation disorders in the critically ill

**Formatted:** Font: Not Bold

46 was highlighted during the COVID-19 pandemic~~The complexity and necessity of~~
47 ~~understanding coagulation disorders in the critically ill became highlighted during COVID-~~
48 ~~19~~ (3)~~.~~ , where the influence of coagulation abnormalities on the prognosis of critically ill

**Formatted:** Font: Not Bold

49 patients was illustrated. This observation illustrates the significance of coagulation’s
50 influence on the prognosis of critically ill patients.
51 Disseminated intravascular coagulopathy is a life-threatening complication of sepsis. Aside
52 from severe sepsis, other diseases can cause DIC, such as malignancy, trauma, liver
53 cirrhosis, snake bites, ~~microorganisms,~~ and obstetric complications. However, sepsis is one
54 of the most common causes of DIC, with DIC being one of the dominant influential factors
55 affecting its outcome (4). The presence of DIC doubles the sepsis mortality rate. During the
56 body’s response to the underlying diseases, there are phases of increased and decreased
57 coagulation characterized by microthrombotic deposits in the capillary flow area or
58 intraparenchymal hemorrhaging in the organ. Thrombotic deposits lead to microinfarction
59 and reduced oxygen supply to the organ, subsequently followed by organ failure with septic
60 shock (5). The pathologic significance of DIC is the simultaneous occurrence of
61 hypercoagulation and hypocoagulation. Developing fibrin deposits increase the
62 consumption~~intake~~ of platelets and coagulation factors, and the simultaneous presence of
63 plasmin causes hyperfibrinolysis (6).
64 In 2001, a uniform DIC assessment was drawn up at the consensus conference of the
65 International Society on Thrombosis and Hemostasis (ISTH). The platelet count, bleeding
66 time in the form of the prothrombin index in %, D-dimer level, and fibrinogen level are
67 among the most important parameters of the ISTH score (7). DIC is also often present in
68 sepsis with multiple organ failure.
69 Challenges to developing treatments are its currently unclear basic etiology and the role of

**Formatted:** Left, Line spacing: Double

70 its interactions with the immune system. Existing animal DIC models can be categorized
71 according to medication-induced (tissue factor, thrombin or snake poison) and infectious
72 variants, such as DIC caused by LPS (lipopolysaccharide) or bacteria. None of these
73 models present a suitable variant that takes into account the complexity of a polymicrobial
74 infection with a reaction by the immune system. Regarding DIC, the effect of the
75 inflammasome on complex blood components, such as platelets, plays a vital role. Recent
76 data imply that the activation of the inflammasome is the decisive factor in mortality (8).
77 The inflammasome introduced by Jurg Tschopp and colleagues in 2002, is a term

**Formatted:** Font: 12 pt

78 explaining a multi protein complex belonging to the innate immune system. Triggered by
79 intracellular NOD-like-receptors (NLRs), detecting danger signals in euraryotic cells of

**Formatted:** Font: 12 pt

80 bacterial origin, it consists of NLR-protein (a family of 22 NLR), an Adapter protein (ASC)

**Formatted:** Font: 12 pt

81 and inflammatory Caspases, e.g. Caspase 1. The inflammatory Caspases where activated by
82 intracellular bacterial products e.g. Flagellin or LPS leading to Pyrocytosis, a form of lytic
83 cell death, causing the release of Interleukin 1beta and Interleukin 18 (9, 10). In previous

**Formatted:** Font: 12 pt

84 studies, our working group was able to demonstrate a strong increase in interleukin 1 beta
85 in the CASP model(11). In Pyrocytosis the plasmamembrane rapidly ruptures and

**Formatted:** Font: 12 pt

86 thrombogenic substances as Tissue Factor are released, which additionally pours coals into
87 the fire of DIC and triggers coagulation cascade(8, 12). Beside this the inflammasome is

**Formatted:** Font: 12 pt

88 currently discussed as misguided immune responses in chronic inflammatory situations,
89 metabolic syndrome, gout and as a reason for islet cell death in type 2 diabetes in literature.

**Formatted:** Font: 12 pt

90 The current literature criticizes the existing artificial DIC models for not fully incorporating
91 the complexity of sepsis (13).
92 The model of colon ascendens stent peritonitis (CASP) is an intra-abdominal sepsis model
93 created by hollow organ perforation. Pursuant to the S3 consensus conference, human
94 sepsis is defined as a life-threatening situation due to a dysregulated organ response to
95 infection (14). The CASP model seems to reflect the organ dysfunction observed in human
96 sepsis, while other animal models are appropriate to reproduce endotoxin shock (LPS
97 model) or to display systemic inflammatory response syndrome (SIRS), such as cecal
98 ligation and puncture (CLP). In these models, dealing with bacterial pathogens, Bacteremia
99 is vital for developing DIC, which is produced in the CASP model by continuous leakage in
100 the area of the cecum.
101 In this paper, we present our comprehensive CASP coagulation analyses and based on our
102 findings, we recommend using the CASP model for the complex analysis of infectious DIC
103 in animal models because CASP, with a ~~high yielding production~~repetitious accuracy, is
104 able to recreate a realistic interaction of the coagulation parameters in the complex situation
105 of severe polymicrobial sepsis.

##### 106 Materials and Methods

107 All experiments were performed on 8- to 10-week-old female C57BL/6 mice. The animals
108 were housed in a temperature-controlled room with a 12-hour light and dark cycle. All
109 experiments were approved by the animal ethics committee of the local animal protection
110 authority (LALLF, State Office for Agriculture, Food Safety and Fisheries Mecklenburg-
111 Western Pomerania; LALLF M-V/TSD/7221.3.-1.1-056/12 and 7221.3-1-056/14.). The
112 results shown were created as part of the animal experiments of these projects. In total, the
113 data from 117 C57Bl/6 mice are shown. Different numbers of animals in the groups are
114 originated by our efforts to keep the control groups as small as possible, if the experimental
115 setting allows us to expect stable results without a wide range of fluctuation. In addition,
116 we considerated that animals in the CASP group could be withdrawn due to excessive
117 stress. In order to avoid small numbers in CASP group, more animals where included, to
118 obtain meaningful results. All efforts were made to minimize the suffering of the mice,
119 enrichments were provided.

120

###### 121 Colon ascendens stent peritonitis (CASP)

122 CASP surgery was carried out according to the method described by Traeger et al. 2010
123 (15). A solution of 0.5 ml xylazine (20 mg/ml), 1.0 ml ketamine (100 mg/ml) and 8.5 ml
124 isotonic NaCl solution was used to anesthetize the mice. By i.p. application of 10 μl
125 anesthetic / g body weight, the animals reached a sufficient depth of anesthesia after about
126 5 minutes. This was checked by the disappearance of pain stimuli on the tail and the
127 inability to trigger leg reflexes. About an hour after application, the animals became lively
128 again and were able to move freely after 2-3 hours.
129 A 16 G cannula (BD Venflon, Franklin Lakes, USA) was introduced 15 mm distal to the
130 ileocecal valve and attached with 7/0 sutures. This stent caused continuous leakage in the
131 area of the cecum and simulated postoperative polymicrobial sepsis. The abdominal wall
132 was closed after repositioning the intestine.

###### 133 Analgesia and Monitoring

134 All mice received 0.1 mg/kg buprenorphine intramuscularly per day to treat pain. Stress
135 scores were collected and evaluated every 6 hours. If the stress severity score (SSS) on the
136 animals exceeded a score of 10, they were killed painlessly under anesthesia by cervical
137 dislocation (16). Some values of n in this study are occasionally caused by animals that
138 were exceeded according to their stress severity score (SSS > 10).

###### 139 Measurement of the platelet count

140 The platelet count was measured in the whole blood of the model vs. the untreated animals.
141 To do so, we used an impedance-based measurement method (VetScan®, Abaxis, Union

**Formatted:** English (United States)

142 City, USA). After 20 hours, the animals’ blood was collected by retro-orbital puncture
143 followed by anticoagulating with EDTA.

###### 144 Measurement of the bleeding time

145 To determine the bleeding time, we clipped off 2 mm of the tail tip with a scalpel and
146 measured the time until the onset of hemostasis, following the method of Dejana et al. (17).
147 The bleeding was made visible by transferring the wounded region into a Falcon tube filled
148 with 15 ml of 0.9% NaCl solution at 37 °C. If hemostasis did not occur within 300 sec, the
149 measurement was stopped, and the value was set to 300 sec. The bleeding time was
150 measured in 3 different experiments done within one month, 23 untreated animals were
151 included and 25 CASP-operated animals, we used animals that where proceeded for further
152 analysis, where the Tail-Tip-bleeding test would not interfere with results.

###### 153 Rotational thromboelastometry

154 Then, 400 µl of citrate-anticoagulated blood from each mouse was added to ex-Tem® S
155 (tissue thromboplastin) corresponding to the manufacturer’s specifications for determining
156 the extrinsic coagulation cascade as a correlate for testing the prothrombin and clotting time
157 (CT) using ROTEM® delta (TEM international GmbH, Munich, Germany). The

**Formatted:** English (United States)

158 ascertained values were plotted against each other. Because the analyzer can only evaluate
159 4 samples at the same time, d~~D~~ata from two ~~experiments~~ experimental days were ~~pooled~~
160 collected to minimize the time between blood sampling and analysis of the probes in
161 ROTEM® delta.

**Formatted:** English (United States)

162 .

###### 163 ELISA

164 D-dimer ELISA
165 To analyze the D-dimer concentration of the septic and untreated mice, plasma at a 1:6

**Formatted:** Font: Bold, English (United States)

**Formatted:** Normal (Web), Space Before: 12 pt, After: 6 pt

**Formatted:** English (United States)

166 dilution was measured with the *Stago* Asserachrom® D-dimer ELISA kit **(**Asni`eres sur

**Formatted:** English (United States)

**Formatted:** English (United States)

167 Seine, France). All steps were carried out according to the manufacturer’s specifications,
168 measuring the OD after terminating the reaction on the ELISA reader at 450 nm. Standard
169 curves and concentrations were prepared and analyzed using GraphPad Prism 8 software.

**Formatted:** Font: Bold

170 Fibrinogen ELISA
171 The enzyme-linked immunosorbent assay (ELISA, by the company *Abnova,*Taipei,

**Formatted:** Font: Not Italic

172 Taiwan) was conducted with an anti-fibrinogen antibody precoated 96-well microtiter plate
173 according to the manufacturer’s specifications. The plate was incubated with diluted mouse
174 plasma (1:30,000) for 10 minutes at room temperature. The plasma was produced from
175 anticoagulated whole mouse blood by centrifugation for 15 minutes at 2,000 g. Reaction
176 detergents were added according to the ELISA manufacturer’s specifications. The optical
177 density was measured using an ELISA microplate reader after terminating the reaction.
178 Standard curves and concentrations were prepared and analyzed using GraphPad Prism 8
179 software.

###### 180 Immunohistological staining

181 The organ cryosections were each incubated with 20 µl 20% FCS buffer for 10 minutes at
182 room temperature and then treated with peroxidase blocking reagent for 10 minutes,
183 followed by washing the sections twice with PBS. For single staining of platelets in the
184 sections of the liver, anti-fibrinogen antibodies were not applied. The primary antibody
185 anti-CD42c FITC (Emfret, Würzburg, Germany) was incubated at 5 µl in 100 µl 20% FCS
186 buffer at 4 °C, the sections were washed again, and then incubated with the secondary
187 antibody Alexa 488 at a dilution of 1:100.
188 We used 100 µl of the antibody mixture for the double staining of fibrinogen/CD42c
189 (CD42c 1:100; fibrinogen 1:50 in PBS/20% FCS), which was incubated at 4 °C. Serving as
190 an isotype control, the second organ section was treated with PBS/20% FCS (the isotype
191 controls are not shown). Subsequently, the sections were incubated with 50 µl of the
192 peroxidase-coupled secondary antibody (1:100). After additional washing, the organ
193 sections were immersed in amplification diluent with 50 µl biotonyl tyramide (1:200), and
194 after washing, 50 µl of anti-FITC-Alexa 488 (1:100) and streptavidin TRITC (1:200) were
195 added in PBS/20% FCS. In the final step, the organ sections were treated with fluoroprep,
196 and after examination with a Keyence Fluorescence Microscope BZ-9000 (Keyence,
197 Osaka, Japan) at 20 x optic, all microscope images were evaluated using the BZ-II
198 Analyzer. The TRITC positive area was analyzed with ImageJ software.
199 **Hematoxylin-eosin (H.E.) staining**
200 The liver removed postmortem was embedded in paraffin-blocks, according to a standard

**Formatted:** Normal

201 protocol, sectioned into 3 µm slices on the microtome, fixed on a slide and stained. Sections
202 were deparaffinized in two consecutive xylene baths for 5 min each. The sections were
203 hydrated using a descending ethanol series (95%, 80%, 70%; 5 min each) and rinsed in
204 double-distilled water. This was followed by incubation in Mayer's hematoxylin solution for
205 15 minutes before rinsing again with double distilled water. Subsequent counterstaining was
206 performed with eosin Y and acetic acid for 4 min followed by rinsing twice. Dehydration
207 was then carried out using an ascending ethanol series (70%, 80%, 90%) and cleaning with
208 two xylene baths for 5 min each. The sections were covered with glycerine gelatin.

**Formatted:** Font: 12 pt

###### 209 Statistical analysis

210 Statistical analysis was calculated with GraphPad Prism version 8.0.0 for Windows,
211 GraphPad Software, San Diego, California USA. When data are shown as significantly
212 different, the p value is two-tailed < 0.05 by a Mann–Whitney test. The group sizes are
213 mentioned in the Results section. All values are marked with dots. The ~~mean~~median values
214 are depicted as bold lines, and the ~~standard deviation~~interquartile Range (IQR~~SD~~) is
215 depicted and mentioned in the legend of the figures (in brackets).

##### 216 Results

###### 217 Platelet count

218 The platelet count in the septic animals dropped very significantly to values well below the
219 normal range (Figure 1). The experiments were carried out repeatedly, and all mice
220 developed thrombocytopenia 20 hours after CASP induction. We were able to show a
221 significant decrease in the platelet count to 370.,000 platelets/µl below the reference value
222 of mouse C57Bl/6 (450.,000–900.,000 platelets/µl) reproducibly occurred 20 hours after
223 inducing sepsis. Counts below 450.,000 platelets/µl were consistently reproducible after
224 CASP surgery, decreasing from 7.,1~~2~~ (1.76 IQR interquartile range~~± 0.68~~ IQR~~SD standard~~
225 ~~deviation~~) × 10^9^ platelets/ml to 3.,14 (~~±~~ 3~~1~~..46~~09~~ IQR~~SD~~) × 10^9^ platelets/ml. On average,
226 the model mice lost approximately 60% of their circulating platelets (p = 0..0016; n = 5
227 untreated group; n = -8 CASP group).
228 Figure 1
229 Fig 1. Platelet count 20 hours after CASP surgery
230 Untreated mice had a mean platelet count of 7.,12 (1.76 IQR~~± 0,68 SD~~) × 10^9^/ml, white column. Mice
231 that underwent CASP surgery showed a reduction to 3.,140 (3.46~~± 1,09~~ IQR~~SD~~) × 10^9^/ml, black column.
232 Septic mice lost 60% of their platelets. The decrease in platelet count was highly significant (p = 0,0016**; n

233	= 5 untreated group; n = ~~–~~ 8 CASP group). The bold black line depicts the median.

###### 234 Tail tip bleeding time

235 We analyzed the bleeding time of septic and untreated mouse tail tips. Septic mice showed
236 a significantly prolonged bleeding time compared to the untreated control animals (Figure
237 2). On average, the median bleeding time of septic animal tail tips was 93~~142.8~~ (260~~± 104~~
238 IQR~~.9 SD~~) seconds. In contrast, untreated animals showed a bleeding time of 57~~62.7~~ (197
239 IQR~~± 39.12 SD~~) seconds. This difference was highly significant (p < 0.001; n = 23
240 untreated group; n = -25 CASP group).
241 Figure 2
242 Fig 2 Tail tip bleeding time in seconds
243 Shown are bleeding times out of the tail tip of 8- to 10-week-old C57Bl/6 mice divided into CASP-
244 operated, black column, and untreated animals, white column, 20 hours after CASP induction exhibiting
245 significant differences in bleeding time (p < 0.001***; n = 23 untreated group; n = -25 CASP group). The tail
246 tip bleeding time was 93 ~~14~~*~~2~~*~~.8~~ (260 IQR~~± 104.9 SD~~) seconds in septic mice and untreated mice showed a
247 bleeding time of 57~~62.7~~ (197 IQR~~± 39.12 SD~~) seconds. The bold black line depicts the median~~an~~.

###### 248 Thromboelastometry

249 Clotting time (CT) – enzyme activity
250 Rotational thromboelastometry (ROTEM) was used to obtain a correlate of the prothrombin
251 time test according to the specifications of the ISTH 2001 DIC criteria. Clotting time (CT)
252 was determined using EXTEM and clot formation time and MCF were also measured (data
253 are not shown due to their similar results to CT). After 20 hours of CASP induction, we
254 detected a prolonged median CT in the model mice of ~~193.6~~109 (409 IQR~~± 171.3 SD~~)
255 seconds, while the untreated control animals showed a CT of 80~~76~~ (21 IQR~~± 8.1 SD~~)
256 seconds. The variances were significantly different. The results are depicted in Figure 3.
257 This difference also proved to be highly reproducible and the p value was p = 0.0043 (n = 6
258 untreated group; n = ~~5-~~5~~6~~ CASP group).
259 Figure 3
260 Fig 3 Clotting time (CT) extrinsic pathway rotational thromboelastometry ROTEM I
261 Illustrated is the CT, measured via rotational thromboelastometry EXTEM in seconds of female 8- to 10-
262 week-old C57Bl/6 mice untreated (white column) or 20 h after CASP surgery (CASP, black column); control
263 mice (untreated) exhibited a stable clotting mechanism within 80 ~~76 s~~ (21 IQR~~± 8.1 SD~~) seconds while 20 h
264 after CASP surgery, the mice showed a prolongation of CT to ~~193.6 s~~109 (409 IQR~~± 171.3 SD~~) seconds (p =
265 0.0043**; n = 6 untreated group; n = 5 CASP group~~5-6~~). The bold black line depicts the median.
266 D-dimer
267 D-dimer levels were also calculated using ELISA. Not surprisingly, 20 h after CASP
268 surgery, there was a significant increase in the blood levels of D-dimer (Fig. 4). The basal
269 D-dimer levels of the untreated animals showed median values of approximately
270 ~~123.0~~120.9 (~~±~~ 66.2 IQR~~25.03 SD~~) ng/ml, while 20 h after CASP surgery, it increased to
271 278.6~~416.7~~ (722.9~~± 267.6 SD~~) ng/ml (p < 0.0001; n = 8 untreated group; n = -11 CASP
272 group).
273 Figure 4
274 Fig 4*.* D-dimer levels in the blood of untreated or CASP-operated C57Bl/6 mice.
275 Statistical results of D-dimer levels 20 h after CASP surgery (CASP, black column) or untreated (white
276 column) 8- to 10-week-old C57Bl/6. Basal blood levels of untreated animals 1203.90 (66.2 IQR~~± 25.03 SD~~)
277 ng/ml increased significantly to 278~~416~~.67 (722.9~~± 267.6 SD~~) ng/ml in CASP-operated mice (p <
278 0.0001****; n = 8 untreated group; n = -11 CASP group); The bold black line depicts the median.
279 Fibrinogen
280 Fibrinogen analysis in the plasma of septic mice was carried out by comparing septic with
281 untreated mice using a sandwich ELISA technique, which revealed a significant decrease
282 (CASP: 29~~50~~.71~~68~~ (126~~± 54~~.3 ~~dg/ml~~ IQR~~SD~~) mg/dl blood plasma vs. untreated: 185.2~~73.4~~
283 (83.91~~± 37.81 mg/dl~~ IQR~~SD~~) mg/dl blood plasma) (p = 0.03, n = 4 untreated group, n = -5
284 CASP group). The results are depicted in Figure 5. These results could be verified by
285 morphological imaging via immunohistochemical staining (Figure 6).
286 Figure 5
287 Fig. 5 Fibrinogen blood levels 20 h after CASP surgery
288 Statistical results of fibrinogen blood levels 20 h after CASP surgery in 8- to 10-week-old C57BL/6
289 mice depicting a significant decrease in fibrinogen plasma levels in CASP-operated mice (CASP, black
290 column). Control animals (untreated, white column) showed ~~173.4~~185.2 (83.91~~± 37.81~~ IQR~~mg/dl SD~~) mg/dl
291 vs. 29.71~~50.68~~ (126.3~~± 54.3 mg/dl~~ IQR~~SD~~) mg/dl blood levels 20 h after CASP surgery (p = 0.03*; n = 4
292 untreated group; n = -5 CASP group). The bold black line depicts the median.
293 Platelet deposits in the liver
294 To demonstrate thrombus formation in shock organs, we conducted HE staining of the liver
295 (spleen and lung but could not find any thrombus formation). To answer the question
296 regarding the fate of the missing platelets, we decided to proceed with further platelet-
297 specific staining of the respective organs and found significant platelet accumulation in the
298 microvascular bed of the liver, as shown in Figure 7. Here, we present representative results
299 and summarize the results in Figure 8. We could clearly demonstrate a significant increase
300 in the accumulation of platelets in the liver.
301 Compared to the untreated control group (p = 0.0002), the liver of C57Bl/6 mice showed a
302 significant increase in the platelet-specific marker CD42c (green dye Figure 8). On average,
303 the control mice had a FITC~~FITC~~-positive area of 0.54~~5~~% (0.6% IQR~~± 0.18% SD~~) (n =
304 10). The medium fluorescence signal in the CASP-operated animals increased to 1.77~~59~~%
305 (1.69~~± 0.65~~% IQR~~SD~~) of the area (n = 7). We could not find any differences in spleen and
306 lung.
307 Figure 6

Fig 6. Frozen sections of the liver with immunohistochemistry staining with an anti-fibrinogen antibody Alexa 488 and platelet anti-CD 42c antibody TRITC. Pictured: left row untreated control animals (a-c) showing liver sections of untreated 8- to 10-week-old C57Bl/6 (3 examples a-c untreated and d-f CASP), the green dye (Alexa 488) represents the fibrinogen amount in the liver sinusoids, the orange dye (TRITC) shows platelet aggregates, the right column is 20 h after CASP surgery (d-f, scale bar 100 µm, negative control not shown).

308 ~~Regarding untreated animals, the double staining of fibrinogen and platelets in CASP~~
309 ~~sepsis clearly verified increased fibrinogen in the area of the sinusoids, thus indicating a~~
310 ~~direct influence on the blood components where fibrinogen seemed to disappear during~~
311 ~~sepsis.~~The double staining of fibrinogen and platelets in CASP sepsis clearly verified
312 increased fibrinogen in the area of the sinusoids in untreated mice, while fibrinogen was less
313 readily seen in those with sepsis.
314 Figure 7

Fig 7. Immunohistochemical staining of frozen liver sections 20 h after CASP with anti-CD42c*FITC/Alexa 488. Depicted are representative results of frozen sections from 8- to 10-week-old C57Bl/6 liver tissue with immunohistochemistry staining of platelets (green dye) with anti-CD 42c antibody*FITC/Alexa 488. The left row shows only a few platelets uniformly distributed in the liver tissue of untreated animals (a), while the right row shows platelet accumulation and aggregates in the liver tissue 20 h after CASP surgery (b); (scale bar 100 µm, negative control not shown, quantification

315 Figure 8
316 Fig 8. Accumulation of hepatic platelets 20 hours after CASP surgery.
317 Pictured is the percentage area of FITC/Alexa 488-positive areas on the fluorescent section in proportion to
318 the entire section. There was a significant increase (p < 0.001***) in the percentage of the FITC/Alexa 488-
319 positive area, i.e., an increased accumulation of hepatic platelets in CASP-operated animals of 1.77~~1.59~~%
320 (1.69% IQR~~± 0.65% SD~~) of the area (n = 7) compared to untreated animals with 0.545% (0.6% IQR~~± 0.18%~~
321 ~~SD~~) of the FITC-positive area (n = 10). The bold black line depicts the median.

##### 322 Discussion

323 DIC is a very complex and dynamic process. The different routine test methods
324 continuously show a point-in-time snapshot of the coagulation situation with coexisting
325 increased coagulation and hyperfibrinolysis. To understand the complexity of this process,
326 it is reasonable to transfer the ISTH 2001 criteria to the murine system for DIC research
327 (13). Admittedly, there are other criteria that are well known, e.g., the Japanese Ministry of
328 Health and Welfare (JMHW) criteria and the Japanese Association for Acute Medicine
329 (JAAM) criteria, each of which has advantages and disadvantages. We applied the ISTH
330 criteria because they are well established(2, 18) and were previously used in other animal
331 studies(13).
332 Differences from the human progression of sepsis have been observed in animal models.
333 The fulminant sepsis of CASP-operated mice triggers hyperinflammation followed by
334 immunosuppression. In the human system, however, the phase of hyperinflammation is
335 often absent, and there is immunosuppression or hypoinflammation instead (19). The
336 different initial immunological situations of mice and humans may also produce variations
337 in the frequency of thrombocytopenia. Only approximately 54% of human septic cases
338 develop thrombocytopenia (20). In our current paper, however, almost all CASP-operated
339 mice developed a significantly lower platelet count after 20 hours (see Fig. 1). It can be
340 assumed that the thrombocytopenia of the mouse is proof of polymicrobial sepsis. The
341 reasons for the high reproducibility of the results are the well-established CASP mouse
342 model (21) and the 16G CASP method used, which presents a model of severe sepsis with a
343 mortality rate of 70% (22). Hence, the first vital aspect of the ISTH score,
344 thrombocytopenia, could be met in CASP. Clinical studies have shown that the extent of
345 thrombocytopenia correlates with a poor survival rate of critically ill patients, thus being a
346 negative predicative factor (23). Various reasons for the decline of the platelets and its
347 consequences for the organism have been frequently discussed and are as yet unanswered.
348 The current information is that thrombocytopenia (below 150,000 Plt~~Trc~~/µl) may
349 develop in humans through the involvement of platelets in the immune response to Gram-
350 positive and Gram-negative bacteria. Platelets, in their role as covercytes, can directly
351 interact with and eliminate bacteria and thus disappear from circulation (24). An additional
352 reason for the platelet decline may be the accumulation of platelets in the liver and spleen
353 (25). Severe sepsis may result in the already mentioned DIC and, by developing
354 microthrombi in the atrial flow area, lead to multiple organ failure.
355 Due to thrombocytopenia being detected in the murine model, we were also interested
356 in the question of whether thrombocytopenia influenced coagulation times. Owing to the
357 fact that a healthy mouse has many more platelets in its circulation than a healthy human, it
358 was questionable whether thrombocytopenia below 450,000 Plt~~Trc~~/µl even had any
359 influence on murine coagulation. This was, however, definitively answered by the
360 prolonged bleeding time of the septic animals compared to the untreated control animals
361 (see Fig. 2).
362 An additional parameter for examining coagulation function is the mentioned
363 prothrombin index in %, also referred to as the “Quick”-value. This value examines the
364 extrinsic coagulation cascade. We analyzed the extrinsic coagulation cascade as an
365 equivalent using rotational thromboelastometry (ROTEM®). To measure the coagulation
366 time, we used thromboplastin with this test kit (tissue factor; ex-Tem®). As a result, we
367 were able to show that the CASP-operated mouse had a longer extrinsic coagulation time
368 (Fig. 3). Admittedly, our data show the results of small groups (n= 6 untreated group; n = 5
369 CASP group~~5-6~~) and present a large IQR~~SD~~ of 409~~171,3~~ s in the CASP-operated group,
370 which is comparatively high relative to the ~~SD~~ IQR of 21~~8,1~~ s in the untreated group. An
371 explanation for the small group sizes is that the ROTEM®-Analyzer can test 4 probes in
372 parallel, but since time is an important factor in analyzing blood clotting, we minimized the
373 time from taking blood samples to analysis; therefore, 2 experimental cycles were
374 performed, and the tests were performed immediately after blood extraction. Due to the
375 septic circulation conditions of the animals, it was not possible to obtain enough blood for
376 multiple samples from any CASP-operated mouse.
377 In hospitals, thromboelastometry is an appropriate tool to rapidly obtain an overview of
378 the performance of patients’ intrinsic and extrinsic coagulation parameters. This method is
379 of particular relevance in surgical transplantation and sepsis therapy (26). Researchers were
380 able to show that the 30-day survival probability was 85.7% among patients with normal
381 ROTEM® parameters. If there was only one change in the measured coagulation
382 parameters, the 30-day survival probability declined to 58.7% (21). This observation is a
383 strong indication of a lack of coagulation factors. Furthermore, additional criteria of the
384 ISTH Consensus Conference 2001 could also be confirmed in our model. There were
385 elevated D-dimer levels as well as decreased fibrinogen levels in the septic animals.
386 With regard to the severe thrombocytopenia in CASP, the question then was what had
387 happened to the platelets missing from the circulation. To answer this question, we
388 prepared frozen immunohistological sections of the liver, lung, and spleen. Our study was
389 able to show that the liver parenchyma developed significant platelet accumulation 20
390 hours after CASP surgery (Fig. 6a-f and 7a, b). In doing so, the septic animals showed
391 rather diffusely distributed platelet aggregations (Fig. 6d-f TRITC orange dye, 7b Alexa
392 488 green dye), while the untreated animals displayed regularly distributed, individual
393 platelets in the parenchyma (Fig. 6a-c, 7a). There were, however, fewer platelets in the area
394 of central veins (Fig. 7b) in septic animals than in untreated control animals. An increase in
395 the pooling of platelets in the liver of septic animals could also be verified by a significant
396 increase in the fluorescent intensity after staining the platelets with CD42c*FITC/Alexa488
397 (Figure 7a, b). The pathological mechanisms of platelet accumulation are the subject of
398 comprehensive analyses in the current literature. Aside from forming agglomerations, there
399 are other explanations for their increased accumulation in the liver tissue. The literature
400 also describes examinations of other models, such as the cecal ligation and puncture (CLP)
401 and the LPS shock model, showing that platelets induce, among other effects, the
402 recruitment of neutrophils into the vascular bed of the liver where they develop aggregates
403 (27). The sinusoids, situated behind these platelet-leukocyte aggregates, are then
404 underperfused. The platelets also add to septic liver cell damage through interactions with
405 neutrophil granulocytes (28). A crucial factor for the accumulation of platelets in the
406 capillary bed of the liver is the thrombocytic adhesion molecule CD62p. Other authors
407 describe the accumulation of platelets as the body’s defense mechanism against increased
408 coagulopathy in the setting of DIC (29). The hepatocyte asialoglycoprotein receptor, also
409 referred to as the Ashwell receptor, was discovered in 2008 (30). It is able to sequester
410 platelets under the influence of a Gram-negative, systemic infection. For this purpose,
411 bacterial neuraminidases help sequester the n-terminal acetyl lactosamine acids of
412 glycoproteins on the host cells to allow their use in their own energy balance (31). Bacteria
413 that have neuraminidases in their repertoire of virulence factors and belong to the typical
414 intestinal flora are, for example, *Bacteroides* spp. and *Escherichia coli*. Hence, the Ashwell
415 receptor might also produce platelet aggregation in the liver of CASP models. However, the
416 exact aggregation pathomechanism was not examined in this research paper.
417 The histological examinations could not identify a significantly increased aggregation
418 of platelets in the spleen. Human autopsies, too, only inconsistently reveal high
419 microthrombosis in patients with a high DIC score (32, 33). The reason for the difficult
420 histological detection of microthrombosis might be the fact that DIC simultaneously causes
421 hyperfibrinolysis, which might have already removed the thromboses that occurred. The
422 chosen method of immunohistochemistry might not have been appropriate for our model to
423 illustrate sequestered platelets in the spleen. It might be that platelet sequestration by
424 splenic macrophages triggered a reaction in the platelets, and the antibody used against
425 CD42c could no longer be retained.
426 Under sepsis, neutrophil platelet aggregates may develop in the capillary flow area of
427 the lung (34). The aggregation of platelets in lungs during abdominal sepsis in humans as
428 well as peritonitis in mice is well documented in the literature (35, 36). The
429 immunohistochemical analyses showed strong variations in the platelet aggregations in the
430 alveolar flow area, thus preventing a conclusive statement.
431 We were able to reconstruct DIC clinical parameters that met the definition of the
432 ISTH 2001 Consensus Conference (7) in the CASP model. There was a significant decrease
433 in the platelet count, the plasma fibrinogen level was significantly reduced, and the
434 bleeding time was significantly prolonged. We were also able to show that this led to
435 platelet aggregation in the liver parenchyma. D-dimer is an additional parameter of DIC.
436 This fibrin degradation product gives information on plasmin activity and fibrinolysis. A
437 study by the University of Zurich showed that an increase in D-dimer above the normal
438 reference value occurred after abdominal surgical interventions. The maximum value was
439 reached on the seventh postoperative day, with the values returning to the reference range
440 only on the twenty-fifth postoperative day (37). Since CASP is a surgical model for
441 inducing severe sepsis, an elevation of D-dimer can be expected 20 hours after CASP
442 surgery. Consequently, the elevated D-dimer level had no significance for assessing DIC in
443 the model used.
444 Thus, we have been able to verify all ISTH 2001 parameters that define DIC, and from
445 our point of view, the CASP model is an appropriate animal model to exemplify sepsis-
446 induced DIC. As a result, DIC can be studied in this complex context, such as in terms of
447 its medication responses, in a highly reproducible animal model.

###### 448 Conclusion

449 The CASP model is appropriate to analyze DIC diagnostic criteria. Currently, there are
450 few appropriate DIC animal models with the majority being created by injecting
451 substances, such as thromboplastin or lipopolysaccharide, to generate increased coagulation
452 (13). These models lack a realistic simulation of severe sepsis. The advantage of the CASP
453 model is its more realistic simulation of polymicrobial sepsis following a major abdominal
454 surgical intervention. Additionally, the model provides the opportunity to analyze the effect
455 of new medications on septic organisms and to detect the influence of coagulopathy on
456 outcomes. All in all the CASP model is suitable to analyze the complexity of DIC in
457 polymicrobial sepsis.
458 Author Contributions:
459 Conceptualization and project design: JvdL; methodology and performance of
460 experiments: JvdL, TK; data evaluation: JvdL, TK; interpretation of data: JvdL, SD, WK,
461 CDH, SK; writing - original draft preparation: Jvdl, SK, SD, CDH and WK; writing -
462 review and editing: all authors critically reviewed the manuscript.
463 **Funding:** This work was financially supported by the German Research Foundation
464 (RTG 840; grants to JvdL). The funders had no role in study design, data collection and
465 analysis, decision to publish, or preparation of the manuscript.
466 **Acknowledgments:** We would like to thank the Institute of Immunology and
467 Transfusion Medicine, Department of Transfusion Medicine, University Medicine
468 Greifswald for their assistance and opportunity to work with ROTEM® delta. Especially, I
469 would like to express my gratitude to and respect for Birgitt Fürll. She was an inspiring and
470 extraordinary human and a dedicated scientist, with a passion for platelets; unfortunately,
471 she passed away.
472 **Conflicts of Interest:** The authors declare that they have no known competing
473 financial interests or personal relationships that could have appeared to influence the work
474 reported in this paper.
475 **References**
476 1.	Iba T, Connors JM, Nagaoka I, Levy JH. Recent advances in the research and management of sepsis-associated DIC.

**Formatted:** Font: Times New Roman, English (United States)

477 Int J Hematol. 2021;113(1):24-33.
478 2.	Levi M. Another step in improving the diagnosis of disseminated intravascular coagulation in sepsis. Critical care
479 (London, England). 2013;17(4):448-.
480 3.	Tang N, Li D, Wang X, Sun Z. Abnormal coagulation parameters are associated with poor prognosis in patients with
481 novel coronavirus pneumonia. J Thromb Haemost. 2020;18(4):844-7.
482 4.	Akca S, Haji-Michael P, de Mendonça A, Suter P, Levi M, Vincent JL. Time course of platelet counts in critically ill
483 patients. Crit Care Med. 2002;30(4):753-6.
484 5.	Rice TW, Wheeler AP. Coagulopathy in critically ill patients: part 1: platelet disorders. Chest. 2009;136(6):1622-30.
485 6.	Samuels JM, Moore HB, Moore EE. Coagulopathy in Severe Sepsis: Interconnectivity of Coagulation and the Immune
486 System. Surg Infect (Larchmt). 2018;19(2):208-15.
487 7.	Taylor FB, Jr., Toh CH, Hoots WK, Wada H, Levi M. Towards definition, clinical and laboratory criteria, and a
488 scoring system for disseminated intravascular coagulation. Thromb Haemost. 2001;86(5):1327-30.
489 8.	Wu C, Lu W, Zhang Y, Zhang G, Shi X, Hisada Y, et al. Inflammasome Activation Triggers Blood Clotting and Host
490 Death through Pyroptosis. Immunity. 2019;50(6):1401-11.e4.

**Formatted:** Font: Times New Roman

**Formatted:** Font: Times New Roman, English (United States)

**Formatted:** Font: Times New Roman

**Formatted:** Font: Times New Roman, English (United States)

**Formatted:** Font: Times New Roman

**Formatted:** Font: Times New Roman, English (United States)

**Formatted:** Font: Times New Roman

**Formatted:** Font: Times New Roman, English (United States)

491 9.	Martinon F, Burns K, Tschopp J. The Inflammasome: A Molecular Platform Triggering Activation of Inflammatory
492 Caspases and Processing of proIL-&#x3b2. Molecular Cell. 2002;10(2):417-26.
493 10. Dagenais M, Skeldon A, Saleh M. The inflammasome: in memory of Dr. Jurg Tschopp. Cell Death Differ.
494 2012;19(1):5-12.
495 11. Maier S, Traeger T, Entleutner M, Westerholt A, Kleist B, Hüser N, et al. Cecal ligation and puncture versus colon
496 ascendens stent peritonitis: two distinct animal models for polymicrobial sepsis. Shock. 2004;21(6):505-11.
497 12. Bergsbaken T, Fink SL, Cookson BT. Pyroptosis: host cell death and inflammation. Nat Rev Microbiol. 2009;7(2):99-
498 109.
499 13. Berthelsen LO, Kristensen AT, Tranholm M. Animal models of DIC and their relevance to human DIC: A systematic
500 review. Thrombosis Research. 2011;128(2):103-16.
501 14. Singer M, Deutschman CS, Seymour CW, Shankar-Hari M, Annane D, Bauer M, et al. The Third International
502 Consensus Definitions for Sepsis and Septic Shock (Sepsis-3). JAMA. 2016;315(8):801-10.
503 15. Au - Traeger T, Au - Koerner P, Au - Kessler W, Au - Cziupka K, Au - Diedrich S, Au - Busemann A, et al. Colon
504 Ascendens Stent Peritonitis (CASP) - a Standardized Model for Polymicrobial Abdominal Sepsis. JoVE. 2010(46):e2299.
505 16. Kiank C, Holtfreter B, Starke A, Mundt A, Wilke C, Schütt C. Stress susceptibility predicts the severity of immune
506 depression and the failure to combat bacterial infections in chronically stressed mice. Brain Behav Immun. 2006;20(4):359-
507 68.
508 17. Dejana E, Callioni A, Quintana A, de Gaetano G. Bleeding time in laboratory animals. II - A comparison of different
509 assay conditions in rats. Thrombosis Research. 1979;15(1):191-7.
510 18. Kobayashi N, Maekawa T, Takada M, Tanaka H, Gonmori H. Criteria for diagnosis of DIC based on the analysis of
511 clinical and laboratory findings in 345 DIC patients collected by the Research Committee on DIC in Japan. Bibl Haematol.
512 1983(49):265-75.
513 19. Koerner P, Westerholt A, Kessler W, Traeger T, Maier S, Heidecke CD. Surgical trauma and postoperative
514 immunosuppression. Chirurg. 2008;79(4):290-+.
515 20. Tsirigotis P, Chondropoulos S, Frantzeskaki F, Stamouli M, Gkirkas K, Bartzeliotou A, et al. Thrombocytopenia in
516 critically ill patients with severe sepsis/septic shock: Prognostic value and association with a distinct serum cytokine profile.
517 Journal of Critical Care. 2016;32:9-15.
518 21. Adamzik M, Langemeier T, Frey UH, Gorlinger K, Saner F, Eggebrecht H, et al. COMPARISON OF
519 THROMBELASTOMETRY WITH SIMPLIFIED ACUTE PHYSIOLOGY SCORE II AND SEQUENTIAL ORGAN
520 FAILURE ASSESSMENT SCORES FOR THE PREDICTION OF 30-DAY SURVIVAL: A COHORT STUDY. Shock.
521 2011;35(4):339-42.
522 22. Traeger T, Koerner P, Kessler W, Cziupka K, Diedrich S, Busemann A, et al. Colon ascendens stent peritonitis
523 (CASP)--a standardized model for polymicrobial abdominal sepsis. J Vis Exp. 2010(46):2299.
524 23. Crowther MA, Cook DJ, Meade MO, Griffith LE, Guyatt GH, Arnold DM, et al. Thrombocytopenia in medical-
525 surgical critically ill patients: prevalence, incidence, and risk factors. J Crit Care. 2005;20(4):348-53.
526 24. White JG. Platelets are covercytes, not phagocytes: uptake of bacteria involves channels of the open canalicular
527 system. Platelets. 2005;16(2):121-31.
528 25. Itoh H, Cicala C, Douglas GJ, Page CP. Platelet Accumulation Induced By Bacterial Endotoxin In Rats. Thrombosis
529 Research. 1996;83(6):405-19.
530 26. Sivula M, Pettila V, Niemi TT, Varpula M, Kuitunen AH. Thromboelastometry in patients with severe sepsis and
531 disseminated intravascular coagulation. Blood Coagulation & Fibrinolysis. 2009;20(6):419-26.
532 27. Singer G, Urakami H, Specian RD, Stokes KY, Granger DN. Platelet recruitment in the murine hepatic
533 microvasculature during experimental sepsis: role of neutrophils. Microcirculation. 2006;13(2):89-97.

**Formatted:** Font: Times New Roman

**Formatted:** Font: Times New Roman, English (United States)

534 28. Yadav SS, Howell DN, Steeber DA, Harland RC, Tedder TF, Clavien PA. P-Selectin mediates reperfusion injury
535 through neutrophil and platelet sequestration in the warm ischemic mouse liver. Hepatology. 1999;29(5):1494-502.
536 29. Grozovsky R, Hoffmeister KM, Falet H. Novel clearance mechanisms of platelets. Curr Opin Hematol.
537 2010;17(6):585-9.
538 30. Grewal PK, Uchiyama S, Ditto D, Varki N, Le DT, Nizet V, et al. The Ashwell receptor mitigates the lethal
539 coagulopathy of sepsis. Nat Med. 2008;14(6):648-55.
540 31. Grewal PK. THE ASHWELL-MORELL RECEPTOR. Methods in Enzymology, Vol 479: Functional Glycomics.
541 Methods in Enzymology. 479. San Diego: Elsevier Academic Press Inc; 2010. p. 223-41.
542 32. Watanabe T, Imamura T, Nakagaki K, Tanaka K. Disseminated Intravascular Coagulation in Autopsy Cases Its
543 Incidence and Clinicopathologic Significance. Pathology - Research and Practice. 1979;165(4):311-22.
544 33. Rizoli S, Nascimento BJ, Key N, Tien HC, Muraca S, Pinto R, et al. Disseminated Intravascular Coagulopathy in the
545 First 24 Hours After Trauma: The Association Between ISTH Score and Anatomopathologic Evidence. The Journal of
546 Trauma and Acute Care Surgery. 2011;71(5):S441-S7 10.1097/TA.0b013e318232e688.
547 34. Welbourn CRB, Young Y. Endotoxin, Septic Shock and acute Lung Injury - Neutrophils, Macrophages and
548 Inflammatory Mediators 1992.
549 35. Asaduzzaman M, Lavasani S, Rahman M, Zhang S, Braun OO, Jeppsson B, et al. Platelets support pulmonary
550 recruitment of neutrophils in abdominal sepsis. Crit Care Med. 2009;37(4):1389-96.
551 36. Kornerup KN, Salmon GP, Pitchford SC, Liu WL, Page CP. Circulating platelet-neutrophil complexes are important
552 for subsequent neutrophil activation and migration. Journal of Applied Physiology. 2010;109(3):758-67.
553 37. Dindo Da, Breitenstein Sa, Hahnloser Da, Seifert Bb, Yakarisik Sa, Asmis LMc, et al. Kinetics of D-dimer after
554 general surgery. Blood Coagulation & Fibrinolysis July. 2009;20(5):347-52.

555

556

557 Supporting Information
558 S1 Appendix
559 S 1. None accumulation of platelets in spleen and lung 20 hours after CASP surgery.
560 Immunohistochemical staining of frozen spleen and lung sections 20 h after CASP with anti-
561 CD42c*FITC/Alexa 488. Depicted are representative results of frozen sections from 8- to 10-week-old
562 C57Bl/6 organ tissue with immunohistochemistry staining of platelets with anti-CD 42c
563 antibody*FITC/Alexa 488. The upper section shows distribution of platelets in spleen tissue in untreated mice
564 (S1a) and CASP operated (S1b), while the lower section shows platelet dye in the lung tissue untreated (S1c)
565 and 20 h after CASP surgery (S1d); (scale bar in µm).

Response to Reviewers

PONE-D-22-09810

Disseminated Intravascular Coagulation (DIC): Old player creates new perspectives on the polymicrobial sepsis model of CASP.

Dear Dr. Elizabeth S. Mayne,

Thank you for sending us a revision of our manuscript. We would also like to thank the reviewers for suggesting improvements to this work. We tried to answer the individual points conscientiously and changed the illustrations accordingly. Unfortunately, the endnote program doesn't play along with the square brackets today. I will try to fix it.

We look forward to your answer. Stay healthy and safe.

Kind regards,

Julia van der Linde Department General Surgery

University medicine Greifswald

Reviewer #1: This manuscript describes the coagulation profile of a mouse model of polymicrobial intra-abdominal sepsis (CASP), and demonstrates potential as a reliable animal model for further research on the pathogenesis and management of disseminated intravascular coagulation. It is overall well written, but requires some modification:

1) The data analysis has largely been performed on very small numbers of mice, and does not appear to be likely to be normally distributed. As such, the mean results reported may be skewed due to the impact of one or two outliers (particularly in the case of the ROTEM data and the bleeding times). Median and interquartile ranges should be reported instead of means and standard deviations. With these results, it will be easier to determine if all of the coagulation parameters are indeed significantly different in the CASP mice.

*Thank you for your comments, we have endeavored to adapt the charts and present them with the median values and interquartile measurements accordingly.*

2) All of the experiments were not performed on all of the mice. This was explained in the discussion, but should also be mentioned in the methods, along with the overall number of mice tested (including the controls).

*We added comments on this in the part of Materials and Methods Line 118-127.*

3) In the introduction, the inflammasome is referred to in line 72. A brief explanation of what the inflammasome is would be of value.

*Thank you for this note. An explanation was added in Line 84-96, in which we noticed that the described pathophysiology of the inflammasome is even more applicable to the CASP model,*

*since there is a massive release of interleukin 1ß, as could be shown in earlier data. (Maier et al 2004.)*

4) In line 82, it is stated that “Bacteremia is vital for developing DIC,”. This is not correct, as many non-infectious causes of DIC are known (as mentioned previously in the introduction).

*In line 94 we added: …” In these models, dealing with bacterial pathogens,…”*

5) In line 86, the meaning of the phrase “with a high yielding production” is unclear.

In line 99 we changed to: *…”repetitious accuracy”…*

6) The manufacturer along with the manufacturers city and country should be specified for all kits and instruments used (including the Vetscan, the ROTEM, and the D-Dimer and Fibrinogen kits). *Changed in Line:*

*128 BD Venflon, Franklin Lakes, USA*

*142 Vetscan Abaxis Union City, USA*

*158 ROTEM (TEM international Gmbh, Munich, Germany)*

*165 Stago Asserachrom (Asnieres sur Seine, France)*

*170 Abnova Taipei, Taiwan 195 Keyence, Osaka, Japan*

7) In line 128-130, the meaning of the following sentence: “Data from two experiments were pooled to minimize the time between blood sampling and analysis of the probes in ROTEM® delta.” is unclear.

*For the coagulation analysis, the time from blood collection to analysis of the samples should be as short as possible, since the ROTEM can only analyze 4 samples at the same time. the results from 2 experimental days were collected and summarized.*

*Linie 159-161: We changed the sentence to the following:…” Because the analyzer can only evaluate 4 samples at the same time, data from two experimental days were collected to minimize the time between blood sampling and analysis of the probes in ROTEM® delta.”…*

8) For all the results, the number of included mice is given as a range (eg n=5-8 or n=23-25).

Why is this? Please explain in the methods.

*Thank you for your advice: The different group sizes are due to the complex methods used. On the one hand, the animals in the CASP-operated group might had a Stress Severity Score of over 10 and where released from the experiments prematurely, on the other hand, in our experience blood sampling is more difficult in septic animals, this fact was taken into account when choosing a larger group size of CASP.*

*The normal coagulation parameters of C57Bl/6 are well known, so that a large control group could be dispensed with, provided that the experimental method itself does not pose large variables.*

*We changed the ranges and explained it separately.*

9) In line 245, “HE staining of the liver” is mentioned. What is this? Please include in the methods.

*Included in Line 203-212 Results are not shown*

10) In line 254, the FITC positive area is reported. How is this calculated? Please include in the methods.

*Line 202: …“The TRITC positive area was analyzed with ImageJ software.”…*

Minor grammatical corrections:

1) In line 16 it is stated that “In surgical ICUs, DIC is mainly caused by abdominal sepsis”.

Please rephrase this as follows: “In surgical ICUs, DIC is frequently caused by abdominal sepsis” **Done**

2) In lines 45-47, the following: “The complexity and necessity of understanding coagulation disorders in the critically ill became highlighted during COVID-19 (3). This observation illustrates the significance of coagulation’s influence on the prognosis of critically ill patients.” Should be rephrased as follows: “The complexity and necessity of understanding coagulation disorders in the critically ill was highlighted during the COVID-19 pandemic (3), where the influence of coagulation abnormalities on the prognosis of critically ill patients was illustrated.” **Done; Line 46-48**
3) In line 50, I suggest removing “micro-organisms” from the list of causes of DIC. **Done; in Line 57**
4) In line 58, please change “increase the intake of platelets” to “increase the consumption of platelets”. **Done; Line 66**
5) The sentence in lines 258-261 should be rephrased as follows: “The double staining of fibrinogen and platelets in CASP sepsis clearly verified increased fibrinogen in the area of the sinusoids in untreated mice, while fibrinogen was less readily seen in those with sepsis.” **Done; in Line 426-428**

Reviewer #2: With this manuscript, the authors showed that the CASP model is suitable for analysing the complexity of DIC in polymicrobial sepsis. The experiments were performed on 8-10 weeks old female C57BL/6 mice, but the number of animals used was not mentioned in the methodology section. Treated and non-treated animals were compared in terms of platelet count, bleeding time, TEG, D-Dimer, Fibrinogen and immunohistological staining for platelets and fibrinogen. Again, the number of blood samples used for these tests were not mentioned.

*We added the exact numbers.*

The authors found that the CASP model caused thrombocytopenia, elongation of bleeding time, reduction in fibrinogen levels and the presence of D-Dimers in plasma. They mentioned that the CASP model seems superior to other models for inducing DIC, however they did not compare the different models directly. They should rather stated that the CASP model is suitable to analyse the complexity of DIC in polymicrobial sepsis.

*Thanks for your comments, we completed in line 590*

*“All in all CASP model is suitable to analyze the complexity of DIC in polymicrobial sepsis.” This paper is furthermore well-written and recommended for publication.*

In Line 573 we added: The funders had no role in study design, data collection and analysis, decision to publish, or preparation of the manuscript.
